# Anecdotes impact medical decisions even when presented with statistical information or decision aids

**DOI:** 10.1186/s41235-024-00577-3

**Published:** 2024-08-26

**Authors:** Emily N. Line, Sara Jaramillo, Micah Goldwater, Zachary Horne

**Affiliations:** 1 Department of Psychology, University of Illinois, Urbana-Champaign, USA; 2 Department of Psychology, University of Pittsburgh, Pittsburgh, USA; 3 School of Psychology, The University of Sydney, Camperdown, Australia; 4 Department of Psychology, University of Edinburgh, Edinburgh, UK

**Keywords:** Anecdotal reasoning, Medical decision making, Health communication, Reproductive health

## Abstract

People are inundated with popular press reports about medical research concerning what is healthy, get advice from doctors, and hear personal anecdotes. How do people integrate conflicting anecdotal and statistical information when making medical decisions? In four experiments (*N* = 4126), we tested how people use conflicting information to judge the efficacy of artificial and real medical treatments. Participants read an anecdote from someone in a clinical trial, or who had undergone a medical treatment previously, for whom the medical treatment was ineffective. We found that reading anecdotes for either artificial or real medical treatments shifted participants’ beliefs about the efficacy of a medical treatment. We observed this result even when the anecdote was uninformative, was paired with an icon array, or when participants were provided with thorough medical decision aids about reproductive health procedures. Our findings highlight the pervasive effect of anecdotes on medical decision making.

**Supplementary Information:**

The online version contains supplementary material available at 10.1186/s41235-024-00577-3.

## Introduction

Making decisions about medical treatments can be a difficult and stress inducing process. When the decision concerns those we love, or those who are vulnerable, the stakes can make even relatively clear decisions seem paralyzing. People are inundated with popular press reports about medical research concerning what is healthy, get advice from doctors, and hear personal anecdotes from friends, relatives, and the media (see, for example, Sillence et al., 2007). How can people make appropriate medical decisions under these conditions? It might seem obvious that people’s beliefs should reflect the scientific consensus, but when our own and our families’ health is at stake, a compelling narrative or personal anecdote can be hard to ignore (Powell et al., 2020).

The difficulty in determining what is best to do is made more difficult to the average information-consumer because of the systematic spread of misinformation. For example, in response and in parallel to the Dobbs v. Jackson decision from the United States Supreme Court, which made abortion no longer a protected right, political groups and state representatives spread misinformation about the efficacy and safety of reproductive health procedures. The Florida Access Network abortion fund reported an increase in pregnant people contacting them with concerns about the safety of abortions, including unwarranted concerns that abortion causes breast cancer or future infertility (Acevedo, 2022). Just as researchers have observed for reproductive health treatments (Kern & Reader, 2022), hesitancy about common and safe medical treatments (e.g., the MMR vaccine) has been found to be at least partially driven by reliance on anecdotal evidence spread through online communities (e.g., Powell et al., 2018).

Outside of medical decision-making contexts, researchers have found that people are capable of correctly integrating statistical information to make informed decisions (e.g., Allen & Preiss, 1997; Allen et al., 2000; Hornikx, 2005), but they may nonetheless improperly attend to irrelevant anecdotal information, particularly when that information is salient and relates to uncertainty and risk (e.g., Allen & Preiss, 1997; Allen et al., 2000; Shen et al., 2015). It has also been established that narratives are more engaging and comprehensive than statistical information alone (Dahlstrom, 2014), but when learning about new scientific information, anecdotal information might interfere with scientific judgments when that information deviates from the available evidence (Rodriguez et al., 2016).

In contrast, it has *not* be determined under what conditions anecdotes that are presented with summary statistics affect medical decision-making, a domain where we would expect anecdotes to be particularly salient to decision makers. Positive anecdotes (that is, anecdotes highlighting the efficacy of a treatment) increase health-promoting behaviors, but we do not know whether these effects outweigh the effects of statistical information alone (Perrier & Martin Ginis, 2017, 2018). There is also evidence that positive anecdotes do not impact perceptions of vaccines or medical screenings (Nyhan et al., 2014; Sheridan et al., 2016; Voorwinden et al., 2017). Likewise, negative anecdotes (those highlighting the inefficacy or side effects of a medical treatment) can reduce people’s intentions to use treatments or screenings, but many of the negative anecdotes used in prior research involve citing extreme side effects of a medical treatment (Scherer et al.,^2016^; Shaffer et al., 2018). Known cognitive biases also likely play a role in how anecdotal information could impact health decisions.

For example, individuals tend to weigh information perceived as unfavorable more heavily than favorable information (Baumeister et al., 2001).

Altogether, when participants are considering a prospective medical treatment it is uncertain whether anecdotes, both positive and negative, are more or as important than clearly presented statistical information.

Beyond considering the artificially simple contrast between the effects of anecdotes and statistical information, real-life medical decision making is not so easy as comparing a summary of a clinical trial with an anecdote that uniformly supports or is opposed to a treatment. Outside of controlled laboratory studies, neither anecdotal information nor statistical information is presented in isolation; people often learn about medical treatments through media outlets (Dobransky & Hargittai, 2012), which will report both the statistical findings of a clinical trial, while also providing the “human-side” of this trial. For instance, media outlets will cite anecdotes of some of the participants who were in the clinical trial to better highlight the outcomes of the trial. In the months following the roll out of the Covid-19 vaccine, there was an increase in anecdotal reports of the vaccine affecting people’s menstrual cycles, a side effect that has since been confirmed empirically (Male, 2021). Thus, to have a comprehensive understanding of the extent of the impact of anecdotal information on medical decision making—whether positive or negative—researchers need to understand the effects of anecdotal reports from trial participants *paired* with statistical information.

There are further aspects of medical decision making that are harder to capture in controlled studies. Not only do healthcare providers cite overall findings from clinical trials to help their patients make informed decisions, but for decisions that are optional or present greater risks for specific populations, patients are also informed about how demographic details (e.g., sex, race, preexisting conditions) can interact with the efficacy or risks of some medical procedures (e.g., patient demographic information for prednisone; Single Care Team, 2022). Because of the additional uncertainty and complexity in making their decisions, healthcare providers often assist patients by also providing them with *decision aids* that include icon arrays to guide them through their choices. Icon arrays are simple displays that convey proportions in an easy-to-understand format (Lewandowsky et al., 2013). Decision aids often take the form of a kind of step-by-step guide to evaluating the pros and cons of a prospective procedure or treatment intended to increase patients’ knowledge about a procedure (Stacey et al., 2017). Thus, to more clearly resolve the impact of anecdotal information on medical decision making, researchers also need to establish the extent of their influence in the presence of additional tools patients would likely be given by their healthcare provider (e.g., how demographics interact with treatments, decision aids which include icon arrays for communicating complex statistical information, and so forth).

Here, we sought to address two questions: First, when people are presented with both statistical summary information about a clinical trial and anecdotes, and the anecdotes are from a patient *in* the trial, how do participants reason on the basis of this information? Do people follow the summary statistics or do anecdotes still affect their judgments? Some research suggests that anecdotes do not impact the integration of statistical evidence in non-medical domains (Hornikx, 2018), but there is less research on this question in the domain of medical decision making, where the stakes can be high and thus anecdotes might may exhibit stronger effects. Second, what impact, if any, does anecdotal information have on participants’ judgments in applied domains and uncertain situations, where participants might seek out additional information and where individual differences and demographic factors are likely to exert an effect?

### The present experiments

We examined the effect of anecdotes on decision making in novel and real-life medical contexts. In Experiments 1a–1c, we investigated how anecdotal information influences people’s interpretation of a (fake) clinical trial describing the efficacy of a medical treatment. By describing an unfamiliar medical treatment, we were able to better control the anecdotal and statistical information we presented to participants. We specifically gave participants fake anecdotes from patients in the clinical study, anecdotes where the information presented is redundant with the statistical information they also received.

In Experiment 1a, we examined the effects of positive anecdotes, statistical information, statistical information paired with positive anecdotes, and statistical information paired with negative anecdotes. We replicated and extended these results in Experiment 1b. We initially predicted that positive anecdotes would increase participants’ beliefs in a treatment’s efficacy, especially when paired with statistical information about the trial. We were also interested in whether a “negative” anecdote about the treatment being ineffective would impact participants’ beliefs about the treatment efficacy (Baumeister et al., 2001). We reasoned that because people are more sensitive to negative than positive information, it was also possible that negative anecdotes would exert a larger effect than positive anecdotes. However, our initial predictions in Experiments 1a and 1b were exploratory. In Experiment 1c, we also tested whether visual aids affected participants’ understanding of the anecdotal and statistical information they read, and had clear confirmatory prediction that an uninformative negative anecdote would effect people’s medical decisions.

In Experiment 2, we examined how anecdotal information impacted decision making in an applied domain about preventative, but optional, reproductive healthcare treatments. Experiment 2 allowed us to test the generality and applicability of the effects of negative anecdotes on medical decisions that we observed in Experiments 1a–1c. Experiment 2 replicated the key findings about negative anecdotes in Experiments 1a–1c.

We investigated how participants made three key decisions which we chose in order to capture a range of decision thresholds: (1) whether participants thought a prospective treatment was effective, (2) whether they would try a prospective treatment, and (3) whether they would give a prospective treatment to their child (or support use of the treatment in children). Research on the psychology of harm draws a clear distinction between moral patients, for example, vulnerable groups like children, rather than “agents,” people who participate in moral actions (e.g., Schein & Gray, 2018). Consequently, we included these thresholds because it is possible negative anecdotal information would selectively impact people’s decisions when reasoning about their child rather than themselves, perhaps because people are risk-averse when making decisions about moral patients. To anticipate the results, we consistently observed an effect of negative anecdotes on all three medical decisions.

## General methods

### Preregistration

We preregistered the data collection plan and predictions for all four experiments. Analysis scripts, full analytic results, and supplementary online materials (SOM) are available on the Open Science Framework at https://osf.io/fcjgd/. Across all four experiments, we conducted a priori power analyses under a significance testing (rather than Bayesian) framework for ease of communication and because we’re unaware of an agreed on set of recommendations for power calculations of Bayesian multivariate cumulative regression models. In Experiments 1a and 1b, we conducted a power analysis to detect a medium effect (Cohen’s *d* = 0.40 for a between-subjects *t*-test, while recruiting additional participants because we expected some people to miss questions checking their attention). This was the smallest effect size of interest during this exploratory set of experiments. (We should note that we realized upon later inspection of our preregistration documents that we mistakenly stated Experiments 1a and 1b were powered to detect a Cohen’s *d* of 0.3 instead of 0.4. This was a typo that unintentionally was copied across preregistration documents). We substantially increased the sample size for both Experiments 1c and 2 (both of which were confirmatory studies), based on the mean effect size observed in meta-meta-analyses of typical social psychological effects (Richard et al., 2003) and observations about differentiating genuine replication failure from replication failure due to insufficient statistical power (Simonsohn, 2015).

We included attention checks in all experiments. We also included a bot check in Experiment 1c where participants had to write a grammatical sentence. See the “Variable Explanations” documents on the Open Science Framework for the attention check questions used in each experiment.

### Analytic approach

We performed Bayesian estimation using the R package brms (Bürkner, 2017). The priors we set for each analysis are on the Open Science Framework.

## Experiments 1a–1c

In Experiments 1a–1c, we first examined whether anecdotes impact people’s beliefs about the efficacy of a medical treatment for less familiar medical treatments. Consequently, in Experiments 1a–1c, we focused on a plausible but relatively unknown medical treatment that people would not have strong beliefs about. Specifically, we examined people’s beliefs about B-12 injections as means for treating chronic headaches.

### Experiment 1a

#### Participants

In Experiment 1a, we recruited 497 participants through Cloud Research recruitment (47% women, *M*_age_ = 38 years old). Cloud Research has created a curated sample of approximately 75,000 participants from Amazon Web Services (AWS; Chandler et al., 2019; Litman et al., 2017). This sample was developed to remove low quality participants, participants with duplicate IP addresses, and prevent participants with suspicious geocodes from participating in studies. It also requires participants’ country location is verified to remove the likelihood of “bots” in the sample. All subsequent experiments used these recruitment features from Cloud Research to ensure data quality.

Participants were paid approximately $6.00 an hour for participating in a five-minute study. Experiment 1a contained four questions checking participants’ attention throughout the survey and a final attention question at the end asking if participants had been distracted during the experiment. If participants did not correctly answer all of these questions, they were excluded. After excluding participants who missed questions checking their attention, 431 participants remained in our sample. Our exclusion criteria were determined a priori and were in accordance with our study preregistration.

#### Procedure

In Experiment 1a, we presented participants with either statistical evidence, anecdotal evidence, or the combination of both types of evidence about a clinical trial testing the effectiveness of B-12 injections on chronic headaches. The study consisted of three parts: a pretest questionnaire in which we asked participants if they have ever used B-12 injections, an intervention, and a posttest questionnaire. After completing this portion of the study, participants completed individual differences measures related to beliefs in medical skepticism, holistic medicine, and finally demographic questions. For brevity, we will not describe our exploratory analyses of these individual differences measures, but the data containing this information is available on the Open Science Framework.

#### Pretest questionnaire

In Experiments 1a–1c, we sought to create a controlled context in which people judged the efficacy of an unfamiliar, but plausible medical treatment. To that end, participants made decisions about B-12 injections as a treatment for headaches. B-12 injections are not a widely used treatment but may nonetheless seem efficacious to people because most people are familiar with B-12 supplements.

In the pretest phase of the study, participants first answered a brief questionnaire examining their familiarity with B-12 injections (Yes/No), then they indicated whether they are currently receiving or have received B-12 injections (Yes/No), and finally they indicated whether they are currently considering receiving B-12 injections as a medical treatment (Yes/No). After responding to these questions, participants were then asked on a five-point Likert scale whether they believe B-12 injections are an effective medical treatment (1 = “Not effective at all”, 5 = “Extremely effective”).

#### Conditions

After completing the B-12 pretest questionnaire, participants were randomly assigned to one of four conditions in a between-subjects design: the Statistics condition, the Positive Anecdote condition, the Statistics + Positive Anecdote condition, or the Statistics + “Negative” Anecdote condition.

In the Statistics condition, participants read a description of a fake clinical trial examining the effects of B-12 injections on patients with chronic headaches. Specifically, participants read that in a clinical trial with 1000 subjects, B-12 injections were 87.3% effective as a medical treatment for chronic headaches. In the Positive Anecdote condition participants did not receive statistical information but were told “Jamie’s [the protagonist in the anecdote] doctor recommended that she participate in a new clinical trial that was examining the effects of B-12 on headaches.” We included information about the doctor’s recommendation as justification for the patient joining the clinical trial. Participants in the Positive Anecdote condition were then were told that Jamie decided to receive B-12 and subsequently experienced a reduction in her symptoms. In the Statistics + Positive Anecdote condition, participants first read the summary statistics demonstrating the efficacy of B-12 injections (that is, the exact material presented in the Statistics condition). They were then told that they would read about the experience of *one of the subjects in the study*, after which they were presented with the anecdote from the Positive Anecdote condition. Thus, the anecdote described is redundant with the statistical summary information participants had already read. Participants in the Statistics + “Negative” Anecdote condition were given the same materials as participants in the Statistics + Positive Anecdote condition, but the anecdote was a self-report of someone in the trial for whom the treatment was ineffective. Participants learned that “Jamie received a B-12 injection and her headaches, lack of energy, and inability to focus persisted.” Critically, however, Jamie was not described as experiencing any side effects as a consequence of her treatment. Rather, the treatment was only described as being ineffective.

We will highlight three key design decisions about Experiments 1a (as well as Experiments 1b–1c): First, the anecdote participants read contained no *new* information in the conditions where it was paired with statistical information. This is because summary statistics already capture the success or failure of B-12 injections in the clinical trial and the anecdote concerns someone who was in the clinical trial. In other words, the anecdote contains no additional information over and above the statistics we provided participants—the anecdote either describes the treatment as effective or ineffective and no other relevant information beyond this. This point is related to a second design decision: Namely, in the Statistics + “Negative” Anecdote condition, B-12 was described as failing as a treatment but not as introducing any unwanted side effects. The inefficacy of the treatment for this patient is already captured in the statistical information about the treatment’s efficacy.

From a rational decision-making perspective, then, the negative anecdote should not affect participants’ interpretation of the statistical information presented to them. Lastly, we did not include a negative anecdote only condition (that is, one in which participants were not provided with data from a clinical trial but were provided with a negative anecdote). By default (Gigerenzer & Gaissmaier, 2011), people do not take medicine they have no reason to think is effective and have some reason to think is ineffective. Consequently, we assumed that participants would not want to try a medical treatment if we only provided them with information it was *ineffective*. In contrast, we did include a positive anecdote only condition in Experiments 1a and 1b. Our rationale was that positive anecdotes about the efficacy of a medicine could make participants overcome the default tendency of not taking action, including taking unfamiliar medicines (e.g., Gigerenzer & Gaissmaier, 2011; Kressel & Chapman, 2007).

#### Posttest questionnaire

After completing the intervention portion of the task, participants completed a posttest questionnaire in which they were asked whether they believed B-12 injections were an effective medical treatment.

One possibility is that when the stakes are high for a given medical decision, people may be more susceptible to anecdotal information leading them to ignore statistical information. To this end, we also included two additional questions in the posttest questionnaire. First, participants were asked how likely it was they would try B-12 injections on a five-point Likert scale. Second, they were asked how likely they were to give B-12 injections to their child (if applicable). It is possible that a negative anecdote would exhibit a stronger negative effect on people’s reasoning about their child compared to themselves because people are more risk averse when it comes to making decisions that impact their children’s health (e.g., Brody et al., 2005; Johnson et al., 2009).

### Results

We predicted that participants in the Statistics + Positive Anecdote condition would be most likely to think that B-12 injections were effective as a treatment for chronic headaches—the positive anecdote would make salient the statistical summary information. This outcome would suggest that health communication experts could include similar positive anecdotes to increase people’s uptake of statistical information (Allen et al., 2000). In contrast, we were unsure whether the Statistics condition or the Positive Anecdote condition would differ from each other, though the Statistics condition objectively contains stronger evidence.

We were particularly interested in how participants would respond to the negative anecdote in the Statistics + Negative Anecdote condition. One possibility is that presenting participants with a negative anecdote could raise the salience of the inefficacy of B-12 injections, a finding which would be compatible with the known tendency to overweight negative information (Baumeister et al., 2001). However, we were unsure to what extent a single negative anecdote could impact people’s use of the statistical summary information, particularly when that anecdote contains no additional information over and above what is contained in the summary statistics.

We tested our predictions by fitting a Bayesian multivariate cumulative regression model regressing B-12 beliefs (i.e., efficacy beliefs, willingness to try B-12, and willingness to give these injections to their children) on Condition (Reference = Statistics condition) and pretest beliefs about the efficacy of B-12. Following the recommendations of (Bürkner, 2018), we modeled pretest as a monotonic effect because of the ordinal nature of this predictor. A comprehensive discussion of the motivation to model ordinal predictors this way can be found in (Bürkner, 2018).

**Fig. 1:**
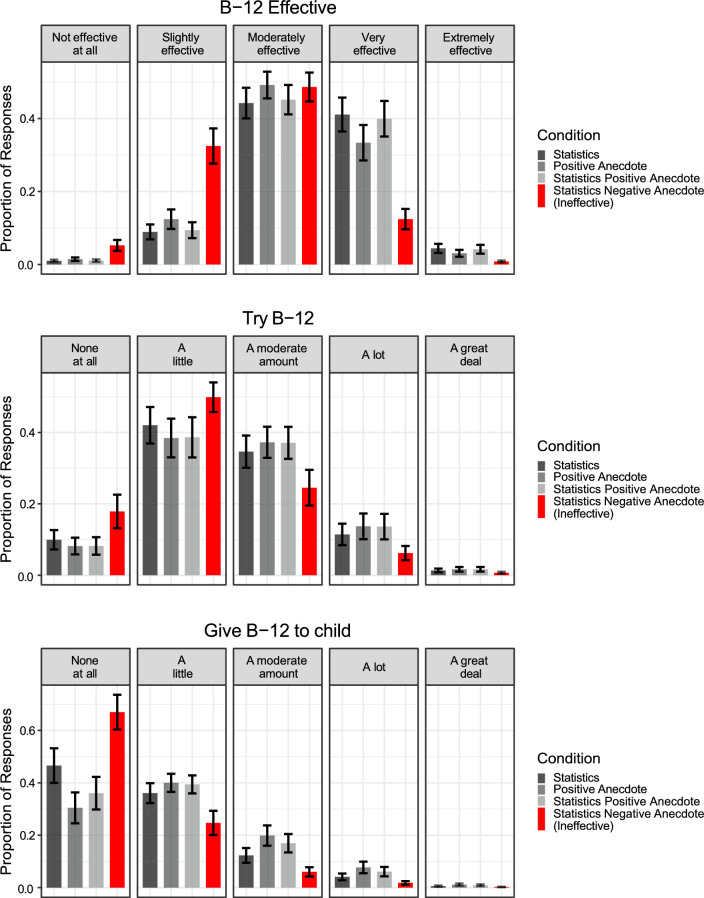
B-12 injection beliefs across conditions in Experiment 1a. The figure indicates that participants in the Statistics + Negative Anecdote condition had less favorable attitudes toward B-12 injections after the intervention relative to participants in the other three conditions. The error bars represent the standard error of model-estimated means. The negative anecdotes are labeled as ineffective in order to differentiate between the anecdotes used in Experiments 1a–1c vs. Experiment 2

This model revealed that the Positive Anecdote, Statistics, and Statistics + Positive Anecdote conditions did not materially differ from each other (see Fig. 1). However, the negative anecdote in the Statistics + Negative Anecdote condition credibly differed from the other conditions, despite the fact that (1) the statistic already summarizes the information contained in the negative anecdote and (2) the negative anecdote in no way suggests that the protagonist suffered a side effect as a result of taking B-12 injections, *b*_Effective_ = − 1.71, 95% CI [− 2.26, − 1.17]; *b*_Try_ = − 0.70, 95% CI [− 1.36, − 0.05]; *b*_Child_ = − 0.87, 95% CI [− 1.55, − 0.20]. A subsequent exploratory analysis interacting pretest beliefs with condition did not improve the model’s fit (see the Supplement).

Altogether, these findings suggest that anecdotal information affected participants’ beliefs. A single positive anecdote carried similar evidential weight as information about a clinical trial, though it appears that it did not affect beliefs additively—the Statistics + Positive Anecdote condition did not differ from the Positive Anecdote condition nor the Statistics condition. (We note however that even in the positive anecdote condition, we described the protagonist as having been in a clinical trial, so participants could reasonably infer her outcome was similar to other people in the trial.)

More worrisome was the effect of the negative anecdote on participants’ reasoning about compelling statistical evidence. One negative anecdote caused people to discount strong statistical evidence, even though the anecdote implied no negative side effects and contained no additional information over and above the information carried by data from the clinical trial.

### Experiment 1b

Experiment 1a suggested that people’s beliefs about the efficacy of B-12 injections are affected by anecdotal information. In Experiment 1b, we sought to further understand the impact of anecdotes on medical decision making in this context. Given that a single negative anecdote may undercut otherwise strong statistical evidence, we sought to explore factors which could reduce the impact of this effect. Consequently, we tested whether presenting participants with *both* a positive and negative anecdote paired with statistical information would lead participants to primarily attend to the statistical information about the efficacy of B-12 injections in treating chronic headaches. Reading contradictory anecdotal information should indicate to participants that a different evidence source (in this case, the statistics) is needed to come to an informed belief about B-12 injections.

#### Participants

We recruited 492 participants through Cloud Research recruitment (50% women, *M*_age_ = 36 years old). Participants were paid approximately $6.00 an hour for participating in a five-minute study. There were three attention checks throughout Experiment 1b, as well as a question at the end asking if participants had been distracted during the experiment. If participants did not pass all four attention questions, they were excluded. After excluding participants who missed attention questions, 431 participants remained in our sample. Our exclusion criteria were determined a priori and were in accordance with our study preregistration.

#### Procedure

The procedure of Experiment 1b was similar to Experiment 1a, with the exception of the conditions participants were assigned to. Namely, we replaced the Positive Anecdote condition with a Statistics + Positive & Negative Anecdotes condition to determine whether including a positive anecdote in conjunction with a negative anecdote would lead participants to focus on summary statistics.

We made two other minor changes in Experiment 1b. First, participants in the Statistics condition were explicitly told *both* the inefficacy and efficacy rates of B-12 injections in treating chronic headaches. We did this to better equate the salience of the inefficacy rate in the Statistics condition to the conditions in which the negative anecdote appeared. Specifically, participants read that “After a two-year trial with 1,000 participants, their study revealed that B-12 injections failed to work for 12.7% of participants and worked for 87.3%.” Second, we changed the Likert scale for our posttest questions regarding the likelihood of trying B-12 injections and giving B-12 to one’s child. These were changed from a five-point Likert scale to a six-point Likert scale which ranged from 1 = “Very unlikely” to 6 = “Very likely” to better reflect the question we asked participants.

### Results

As in Experiment 1a, we made the exploratory prediction that participants in the Statistics + Positive Anecdote condition would tend to have the most positive beliefs toward B-12 injections. We speculated it was possible that when participants in the Statistics condition are explicitly presented with the rate of ineffectiveness, this would raise the salience of the inefficacy of B-12 injections. In turn, this may reduce overall endorsement of the efficacy of B-12 injections relative to the Statistics + Positive Anecdote condition. Both of these predictions were exploratory, as indicated in our preregistration.

Finally, we sought to examine whether inclusion of the positive anecdote with the negative anecdote in the Statistics + Positive & Negative Anecdotes condition would cause participants to primarily attend to the statistical information they received. However, we suspected that the presence of the positive anecdote would not entirely undercut the effect of the negative anecdote on participants’ decisions.

**Fig. 2:**
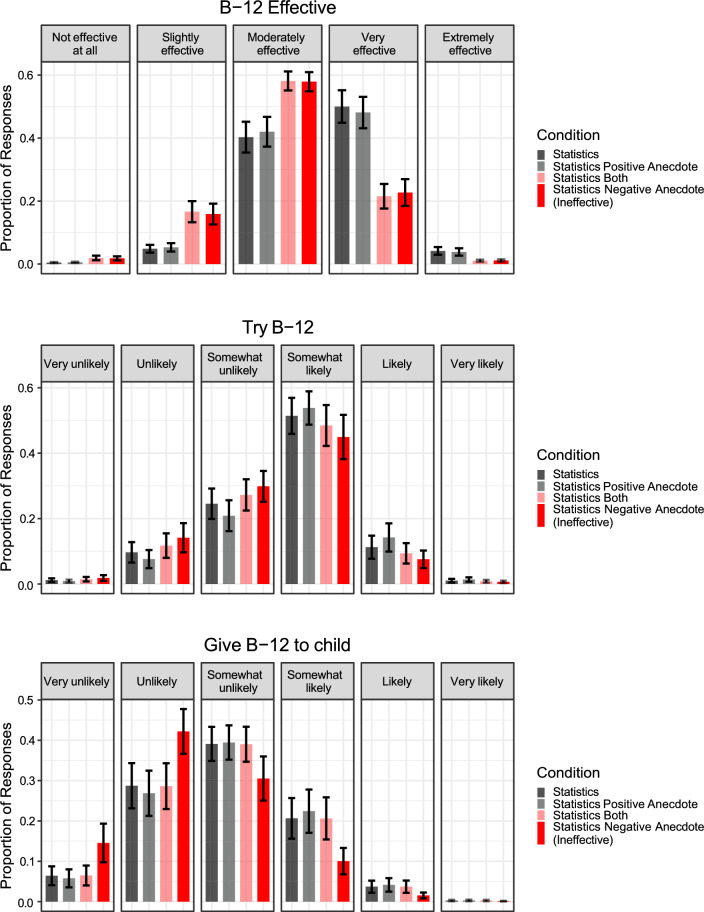
B-12 injection beliefs across conditions in Experiment 1b. The figure indicates that participants in the Statistics + Negative Anecdote and Statistics + Both Anecdotes conditions had less favorable attitudes toward B-12 injections after the intervention relative to participants in the other conditions. The error bars represent the standard error of the model-estimated means. The negative anecdotes are labeled as ineffective in order to differentiate between the anecdotes used in Experiments 1a–1c vs. Experiment 2

We fit a Bayesian multivariate cumulative regression model regressing B-12 attitudes on Condition (Reference = Statistics condition) and Pretest beliefs. This analysis replicated the effects of Experiment 1a (Fig. 2), showing that (1) the Statistics and Statistics + Positive Anecdote conditions did not differ from each other and (2) that participants in the Statistics + Negative Anecdote condition were more likely to discount the statistical evidence from the clinical trial (see Fig. 2), *b*_Effective_ = *−*1.33, 95% CI [− 1.86, − 0.77]; *b*_Try_ = − 0.43, 95% CI [− 1.22, 0.33]; *b*_Child_ = *−*0.91, 95% CI [− 1.72, − 0.17]. The positive anecdote in the Statistics + Positive and Negative Anecdotes condition, however, did not consistently improve participants’ integration of the statistical information, and in some cases, did not differ at all from when participants only received the negative anecdote (see Fig. 2).

### Experiment 1c

A limitation of the prior two experiments is that the error in the estimated effects of positive and negative anecdotes suggested that our sample sizes may be too small to reliably detect the effect of short anecdotes on medical decision making (e.g., Button et al., 2013). We addressed this issue in Experiment 1c by recruiting substantially more participants.

Experiment 1c addressed two questions. First, we addressed the possibility that participants did not realize the anecdote they read was about a person in the study. Our hope was that by visually showing participants that the anecdote they read was about a person in the study we could rule out the possibility that a negative anecdote had its effects just in virtue of it being perceived as *new*, “negatively”-valenced information.

Second, inspired by recent work on data visualization (Lewandowsky et al., 2013; Nyhan & Reifler, 2018), Experiment 1c tested whether the effect of the negative anecdote would persist even when a visual aid was presented to increase understanding of the statistical information participants were presented with. Several recent studies suggest that icon arrays, for instance, can improve understanding of scientific consensus (Lewandowsky et al., 2013; Nyhan & Reifler, 2018). Experiment 1c tested whether the effect of the negative anecdote would persist even when an icon array was used to succinctly express the statistical summary information about the clinical trial.

#### Participants

We recruited more participants in Experiment 1c to ensure that any failure to find an effect of icon arrays on reducing the effect of the negative anecdote was not due to insufficient statistical power. We recruited 1622 participants through Cloud Research recruitment (54% women, *M*_age_ = 38). Participants were paid approximately $6.00 an hour for participating in a five-minute study. There were three attention checks throughout Experiment 1c and a final attention question at the end asking if participants had been distracted during the experiment. If participants failed any of these attention questions, they were excluded. After excluding participants who missed attention questions, 1539 participants remained in our sample. Our exclusion criteria were determined a priori and were in accordance with our study preregistration.

#### Procedure

The procedure of Experiment 1c was similar to that of Experiments 1a and 1b. Participants were randomly assigned to one of four conditions in a 2 (Icon Array: Present or Absent) × 2 (Negative Anecdote: Present or Absent) between-subjects design. All four conditions included the summary statistical information from the Statistics condition in Experiment 1a, allowing us to internally replicate our results in a larger sample.

**Fig. 3:**
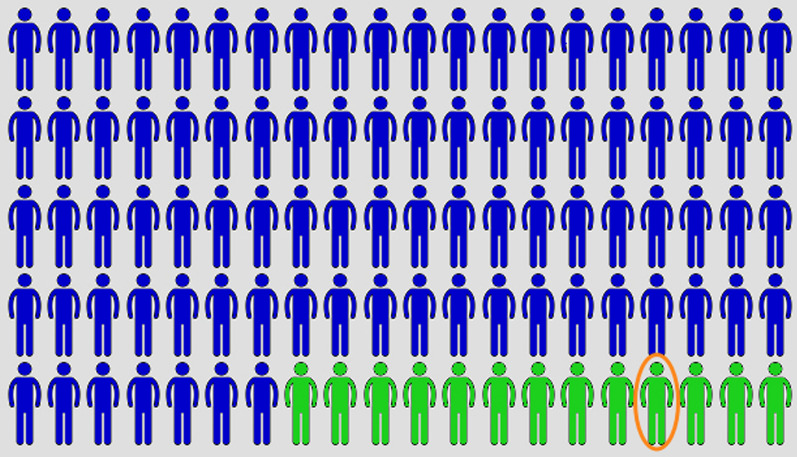
The icon array used in the Icon Array + Negative Anecdote condition in Experiment 3. For the Icon Array only condition, the same array was presented, but without the orange circle

In the Icon Array only condition, participants first read the statistic about the efficacy of B-12 injections as a medical treatment. They were then shown an icon array showing the success rate of B-12 in 100 people (Fig. 3). Participants were then told:

This image is a depiction of the effectiveness of B-12 as a medical treatment. Imagine 100 people received B-12 injections. The blue figures represent participants that would benefit from the B-12 injections. The green figures represent participants who would fail to benefit from the B-12 injections

We chose a “neutral” color scheme and did not include red icons to avoid any immediate associations that might indicate that patients in the clinical trial experienced negative side effects of the treatment. Again, negative anecdotes simply indicated a treatment was ineffective rather than that it had unwanted side effects.

In the Icon Array + Negative Anecdote condition, participants received the same information as the Icon Array only condition but were then told they would read about the experience of one of the subjects in the study and an icon array was displayed with one of the participants circled (Fig. 3), indicating that the anecdote was from someone who participated in the clinical trial.

### Results

We predicted that we would replicate the effect of negative anecdotes on participants’ acceptance of strong statistical evidence, as we found in Experiments 1a and 1b. We also (tentatively) predicted that in the Icon Array + Negative Anecdote condition, the presence of the icon array could weaken the effect of the negative anecdote (indicating an Icon × Anecdote interaction).

**Fig. 4:**
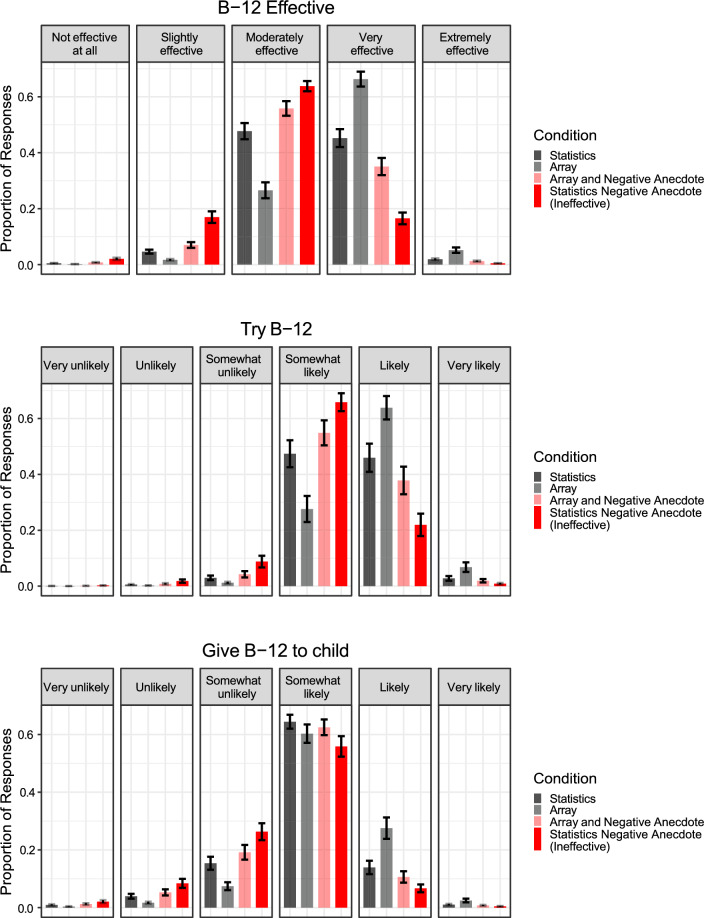
B-12 injection beliefs across conditions in Experiment 1c. The figure indicates that participants in the presence of an anecdote shifted beliefs about B-12 injections in both the condition in which the icon array was present and omitted. The error bars represent the standard error of model-estimated means. The negative anecdotes are labeled as ineffective in order to differentiate between the anecdotes used in Experiments 1a–1c vs. Experiment 2

We fit a Bayesian multivariate cumulative regression model regressing B-12 attitudes on the interaction between Icon Array (Reference = No Array) and Anecdote (Reference = Statistics Only condition), controlling for pretest beliefs. We replicated the effects of Experiments 1a and 1b (Fig. 4), showing that a negative anecdote affected participants’ integration of statistical information, *b*_Effective_ = − 1.49, 95% CI [− 1.83, − 1.15]; *b*_Try_ = − 1.23, 95% CI [− 1.77, *−*0.71]; *b*_Child_ = − 0.87, 95% CI [− 1.31, − 0.44]. Consistent with prior work, we also found that providing an icon array improved people’s integration of statistical information (*b*_Effective_ = 1.02, 95% CI [0.69, 1.35]; *b*_Try_ = 0.89, 95% CI [0.37, 1.40]; *b*_Child_ = 0.86, 95% CI [0.43, 1.27]). Contrary to our tentative prediction, these factors did not interact: *b*_Effective_ = 0.00, 95% CI [− 0.46, 0.47]; *b*_Try_ = − 0.09, 95% CI [− 0.82, 0.59]; *b*_Child_ = − 0.34, 95% CI [− 0.93, 0.22]. The absence of an interaction between both factors indicates that the negative anecdote impacts people’s reasoning even when an icon array is present.

We conducted a series of exploratory analyses examining how incorporating individual differences measures in our models would affect the results, including people’s beliefs about holistic medicine, the reliability of results produced by pharmaceutical companies, participant age, and participant gender. We did not observe any reliable interactions between these predictors and negative anecdotes. These analyses are in the Supplement.

### Summary of Experiments 1a–1c

In Experiments 1a–1c, we tested how people reason about a novel medical treatment when provided with statistical and anecdotal information. In Experiment 1a, we found that a negative anecdote caused people to ignore strong statistical information even though the anecdote described no negative side effects—indeed, the information presented in the anecdote was already captured by the summary statistics presented to participants. In Experiment 1b, we explored whether providing a positive anecdote in addition to a negative anecdote would counteract the effect of the negative anecdote. We found that emphasizing a positive outcome of a clinical trial did not consistently undo the effect of the negative anecdote, again demonstrating the consistency of the effect of negative anecdotes on medical decisions. In Experiment 1c, we found that introducing icon arrays improved integration of statistical information overall, but even in this case, anecdotal information impacted people’s beliefs. This suggests that a single negative anecdote can carry substantial weight when making medical decisions, consistent with prior research (Scherer et al., 2016; Shaffer et al., 2018). However, our findings go beyond prior research by establishing that negative anecdotes do not need to have evidential value to effect people’s decisions.

It is striking that a “negative” anecdote led people to discount strong summary statistics even though the patient was described as suffering no negative side effects because of their treatment. In reality, many medical treatments involve an element of risk, and some treatments can even involve severe side effects. In these situations, anecdotes that contain new information and highlight side effects would, if anything, yield a larger negative impact on people’s ability to properly integrate statistical information (see Shen et al., 2015). Still, one concern with the design of Experiments 1a–1c is the possibility that conversational pragmatics (Clark et al., 1977; Grice, 1975; Schwarz, 1994) induced task demands that led participants to use the anecdotal information we provided them, even though participants would not be affected by this kind of information in less contrived settings. In everyday conversations, people provide as much information as required; if people are given information they assume it must be relevant to the situation at hand (Grice, 1975). One possibility then is that because we provided participants with anecdotes at all—whatever their form—they used them in their decision making not because of some special epistemic status of negative anecdotes but rather because we provided them with this information in the first place.

Our findings cannot be explained by this deflationary alternative hypothesis. For example, we found that providing participants with a positive anecdote did not affect their attitudes toward a treatment when the anecdote was paired with statistical information. We directly replicated this effect in Experiment 1b. In contrast, negative anecdotal information persistently affected people’s integration of statistical information across all three experiments. Nonetheless, in Experiment 2, we addressed this concern more directly in our experimental design.

To examine the breadth of the effects of negative anecdotes on medical decision making, Experiment 2 tested how they impact medical decision making about widely known reproductive health treatments. Testing the effect of anecdotal information in this domain allows us to examine how anecdotal information interacts with demographic information (e.g., sex). Further, as we will detail, turning to real medical decisions allowed us to study how the presence of further information often provided to patients to guide their medical decisions may attenuate the affects of anecdotal information.

Compared to the how much research has been conducted on the effects of anecdotal reasoning and misinformation in, for example, politics, there are very little data on the impact of misinformation and related effects on people’s understanding of reproductive health treatments. Yet, there is substantial amounts of misinformation about women’s health options (e.g., HPV vaccine and mammograms; Nagler et al., 2015), misinformation which may now play a role in the ongoing assault on women’s reproductive rights. This led us to focus on women’s health treatments as an applied context where we could closely examine the impact of ecologically valid anecdotes on medical decision making.

## Experiment 2

People are not typically presented with medical information in an isolated manner about a treatment about which they have little information. Medical practitioners will often provide their patients with decision aids, and patients themselves will often learn about prospective treatments online or by talking to friends and family. A study examining the effects of Google search on doctor-patient relations found two-thirds of patients looked up their symptoms online prior to a doctor’s consultation (Van Riel et al., 2017); patients no longer solely rely on their doctors’ opinions when making medical decisions. Consequently, in Experiment 2, we tested whether negative anecdotes would continue to impact participants beliefs when people receive both decision aids and negative anecdotes, and participants were presented with anecdotes *prior* to being given a decision aid (i.e., not immediately before making their decision).

We used existing medical decision aids and anecdotal information from online discussions where people shared their experiences about a particular medical treatment. Medical decision aids are communication devices developed to provide patients with detailed information about how health procedures occur and the options they have in the administration of these health procedures. Decision aids are often provided as pamphlets in doctors’ offices and include information such as drug or treatment facts, benefits, and potential side effects. We found three preventative women’s health treatment decision aids (HPV testing, Epidurals, and the Hormonal IUD) from The Ottawa Hospital Research Institute and South Carolina Department of Health and Environmental Control. We provide further details about the decision aids in the materials section below.

The anecdotal information was taken from online discussion forums and from existing decision-aid materials (Healthwise Staff, 2022; Kantartist, n.d.; Waller et al., 2007). In these anecdotes, people share their experiences after undergoing a medical treatment. We selected anecdotes that were representative of common anecdotes in terms of length, content, and emotional language. Emotional language impacts the uptake of anecdotal information (Freling et al., 2020), so an inherent limitation of our design is that it relies on real anecdotes which vary in the amount of emotional language they use. Thus, although Experiment 2 is a more ecologically valid test of the effect of negative anecdotes on decision making, it must be acknowledged that we lose the ability to precisely manipulate the information participants read.

### Participants

We recruited 1580 participants through Cloud Research recruitment (53% women, *M*_age_ = 40). Participants were paid approximately $6.00 per hour for participating in a 20 min study. There were four attention check questions throughout Experiment 2 and a final attention question asking participants if they had been distracted during the experiment. If participants failed any of these five attention questions, they were excluded. After excluding participants who missed attention check questions, 1408 participants remained in our sample. Our exclusion criteria were determined a priori and were in accordance with our study preregistration.

We collected the data from Experiment 2 a year before the Dobbs v. Jackson decision was handed down by the United States Supreme Court, so the heightened discussion following this decision could not impact the results of our study.

### Procedure

We tested the effect of negative anecdotes on decision making in contexts that highlighted two key features. First, we aimed to capture the optional nature of some treatments—one could continue to live without the treatment, so a strong test of the effect of anecdotes on decisions should reflect this fact. Second, we selected treatments that are highly effective and thus reflected similar efficacy rates as Experiments 1a–1c. We focused on three medical treatments in reproductive health: the Human Papillomavirus (HPV) test, epidurals as a method of birth pain relief, and the hormonal intrauterine device (IUD). (While the HPV test is not a treatment, it is a preventative measure that would lead participants to receive extensive treatment depending on the results of the test. For simplicity, we refer to these medical contexts as “treatments” throughout the remainder of the paper). Participants were asked about three different treatments (rather than a single treatment—B-12 injections), so we could test the affect of anecdotes across a wider variety of treatments in the event that, unbeknownst to us, one treatment was more polarizing or idiosyncratic than we anticipated.

The procedure of Experiment 2 was similar to that of Experiment 1c: Before the experimental manipulation, participants were asked to complete a pretest questionnaire examining their familiarity about each of the three reproductive health treatments (Yes/No). After responding to these pretest questions, participants were then asked on a five-point Likert scale whether they believe these were effective preventative health treatments (1 = “Not effective at all”, 5 = “Extremely effective”). Following the recommendations of Preston and Colman (2000), we measured participants’ judgments using a five-point Likert scale so that participants could indicate they were unsure about a treatment rather than being forced to either somewhat disagree or somewhat agree with a statement, a possible limitation of our prior experiments. This scale would thus be appropriately “expressive” for the mental representations we would expect participants to have in high-stakes contexts that involve considering complex statistical and anecdotal information.

Participants were then randomly assigned in a 2 (Icon Array: Present or Absent—between-subjects) × 2 (Negative Anecdote: Present or Absent—between-subjects) × 3 (Medical Decision—within-subjects) mixed-effects design. After assignment to one of the four between-subjects conditions, participants read three different vignettes about reproductive health treatments. Vignettes were blocked so that posttest measures were administered after a given vignette (e.g., the IUD posttest questionnaire was administered after the IUD materials). We presented decision aids across multiple pages because of the length of comprehensiveness of the information provided in them. To ensure participants actually considered the content of these aids, we required participants to spend at least 8–15 s on each page before advancing to the next page, depending on the amount of content on the page. As before, all four between-subjects conditions included summary statistical information. The order of the three within-subjects conditions was randomized.

### Conditions

The Statistics Only condition is displayed in the left-most column of Table 1. Here, participants read information from a medical pamphlet about hormonal IUDs (South Carolina Department of Health and Environmental Control, n.d.).

**Table 1:**
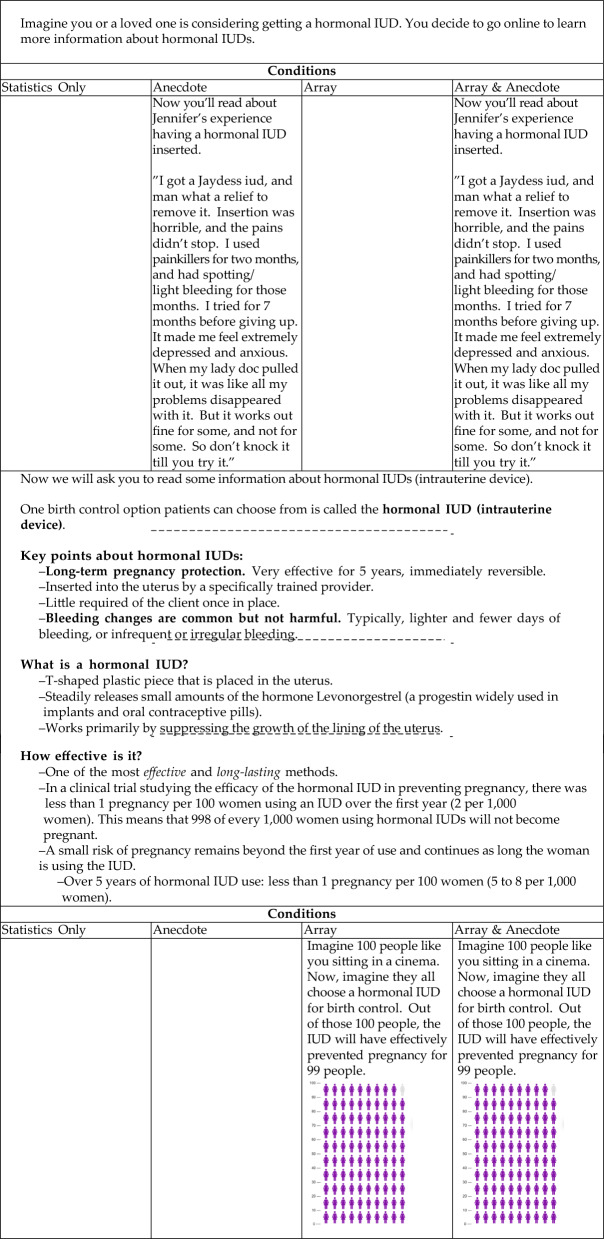

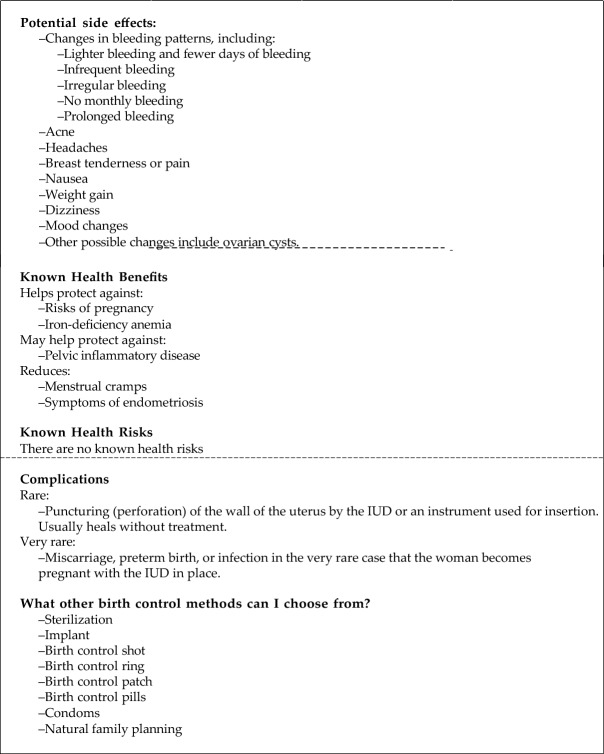
The stimuli for the Hormonal IUD vignette in Experiment 2

This table shows the materials in the order they were presented to participants. The columns indicate materials that are unique to a given condition. For instance, in the Statistics Only condition, the left-most column is empty indicating no additional information was given. When we move to the right to the Anecdote column, we see that before reading the rest of the information about IUDs, participants read an anecdote from someone named Jennifer. These materials were presented to participants over several pages. Page breaks are indicated by the different table sections and dotted lines.

Participants were presented with the following statistical information,

In a clinical trial studying the efficacy of the hormonal IUD in preventing pregnancy, there was less than 1 pregnancy per 100 women using an IUD over the first year (two per 1000 women). This means that 998 of every 1000 women using hormonal IUDs will not become pregnant.

In the Anecdote condition, participants read the same statistical information as participants in the Statistics Only condition. They also read about the experience of Jennifer who had a hormonal IUD inserted. Unlike studies 1a–1c, we presented the anecdotal information at the start of the vignette (i.e., before participants read all of the information in the decision aid). This design decision was motivated by two considerations: First, anecdotal information is comparatively easy for patients to access relative to decision aids and the finding that patients tend to Google experiences of their possible treatments prior to consulting with their physician (Van Riel et al., 2017). Second, providing anecdotal information immediately before asking participants to make a judgment could impose task demands on them (Grice, 1975), a possibility we wanted to eliminate in Experiment 2. In the prior three experiments, we provided participants with anecdotal information after they read about data from a clinical trial and immediately prior to the posttest questionnaire.

When presented with the statistical summary, participants were told that “Jennifer, the patient you read about who chose to have a hormonal IUD inserted, was a patient in this clinical trial.”

**Fig. 5:**
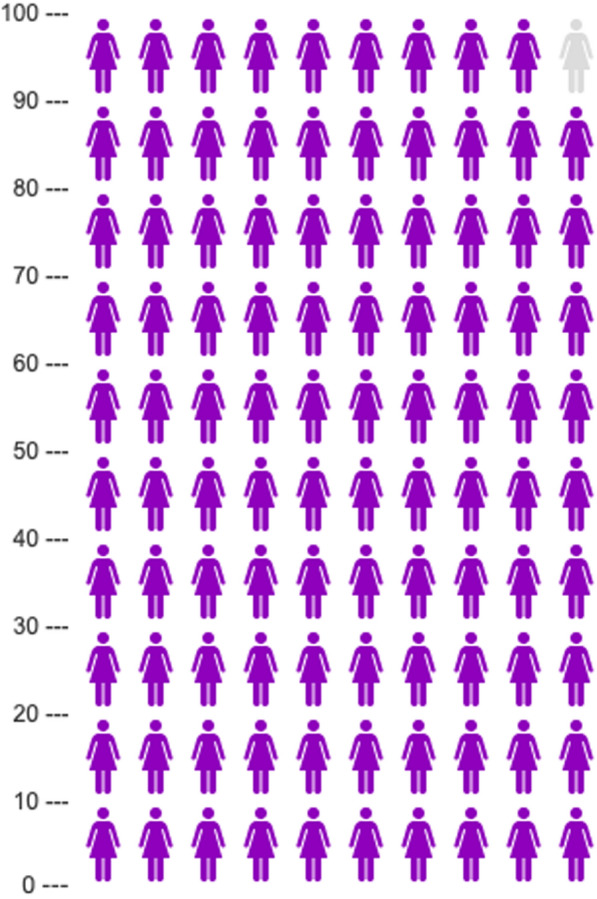
The icon array used in the Icon Array + Negative Anecdote condition for the hormonal IUD vignette in Experiment 3. See SOM for the icon arrays used in the HPV test vignette and the Epidural vignette

In the Icon Array only condition, participants first read a statistic about the efficacy of a medical treatment, such as the hormonal IUD to prevent pregnancy. These vignettes were taken from preexisting medical decision aids (see Table 2). Participants were then shown an icon array showing the success rate of the hormonal IUD to prevent pregnancy (Fig. 5). Participants were then told:

Imagine 100 people like you sitting in a cinema. Now, imagine they all choose a hormonal IUD for birth control. Out of those 100 people, the IUD will have effectively prevented pregnancy for 99 people.

**Table 2:** The anecdotes used in each experiment

Experiment(s)	Treatment	Type	Anecdote
1a, 1b*, 1c	B-12 injections for headaches	Positive	Jamie received a B12 injection and experienced that she had a reduction in the number of her headaches. She was also more focused and had more energy. Because she had more energy and focus, Jamie was able to play with her daughter and read to her every night
1a, 1b*, 1c	B-12 injections for headaches	Negative	Jamie received a B12 injection and her headaches, lack of energy, and inability to focus persisted. This frus-trated Jamie as she was unable to play with her daughter or read to her because of her symptoms
2	Epidural	Negative	Now you’ll read about Jessica’s experience receiving an epidural. “I didn’t really think too much about how I was going to handle labor pain. When I was in the middle of labor, they told me I could have an epidural, and I just said yes. I didn’t like it at all. I couldn’t feel enough to push. Then, I had a bad headache for days afterwards. Of course, it only matters that my baby is healthy, but I won’t have an epidural again”
2	HPV	Negative	Now you’ll read about Emily’s experience receiving the HPV test. “I was quite annoyed, couldn’t really believe it that it happened to me. And because it was sexually transmitted, and because I haven’t had a lot of partners. Yeah wasn’t very happy at all really….I phoned the helpline and they reassured me, they really did reassure me so I felt quite happy after talking with them. I would say it [the anxiety] lessened over the year because of the chat I had with the helpline. I thought well if I hadn’t taken part in that test I would never have known I had it and I knew I was going to have further tests”
2	IUD	Negative	Now you’ll read about Jennifer’s experience having a hormonal IUD inserted. “I got a Jaydess iud, and man what a relief to remove it. Insertion was horrible, and the pains didn’t stop. I used painkillers for 2 months, and had spotting/light bleeding for those months. I tried for 7 months before giving up. It made me feel extremely depressed and anxious. When my lady doc pulled it out, it was like all my problems disappeared with it. But it works out fine for some, and not for some. So don’t knock it till you try it”

Experiments 1a–1c used the same anecdotes. See the supplemental materials for the full text, including the explanation leading up to why Jamie sought out treatment. *Experiment 1b also had a version of this anecdote with a male protagonist. The full materials can be viewed on OSF. The anecdotes from Experiment 2 come from online discussion forums and existing decision-aid materials (Healthwise Staff, 2022; kantartist, n.d.; Waller et al., 2007)

In the Icon Array + Negative Anecdote condition, participants received the same information as the Icon Array only condition but first read about the experience of someone who experienced having a hormonal IUD inserted. After the trial, participants answered questions to test how effective they thought the medical treatments are and whether they would try them themselves or recommend someone they know to try them. Finally, participants who identified as female answered a battery of demographic questions related to reproductive health of female identifying participants. Thus, participants who identify as male were not asked to complete this questionnaire.

We predicted that we would replicate the effect of the negative anecdote on medical decisions that we observed in Experiments 1a–1c.

### Results

**Fig. 6:**
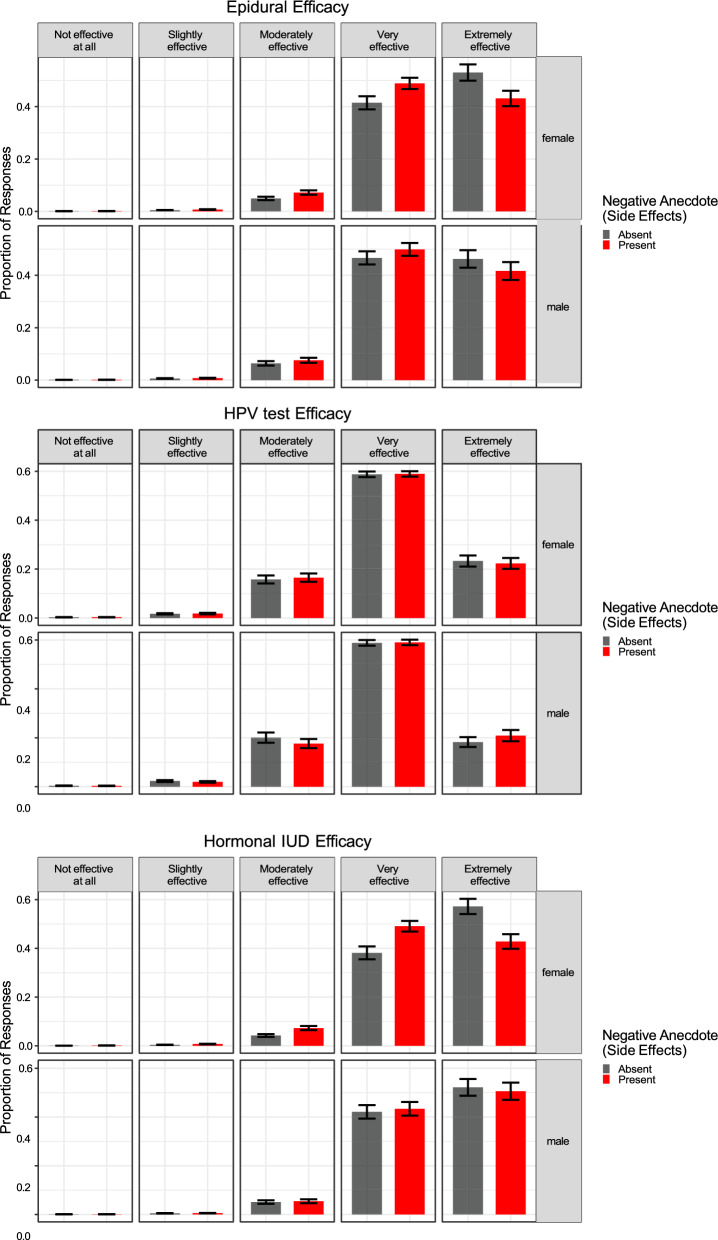
Efficacy beliefs across conditions in Experiment 2. Higher Likert scale choices indicate more favorable attitudes toward health topics. The figure indicates that participants presented with negative anecdotal evidence had less favorable attitudes toward treatments after the intervention relative to participants who were not presented with negative anecdotal evidence. The error bars represent standard errors of model-estimated means. The negative anecdotes are labeled as having side effects in order to differentiate between the anecdotes used in Experiments 1a–1c vs. Experiment 2

**Fig. 7:**
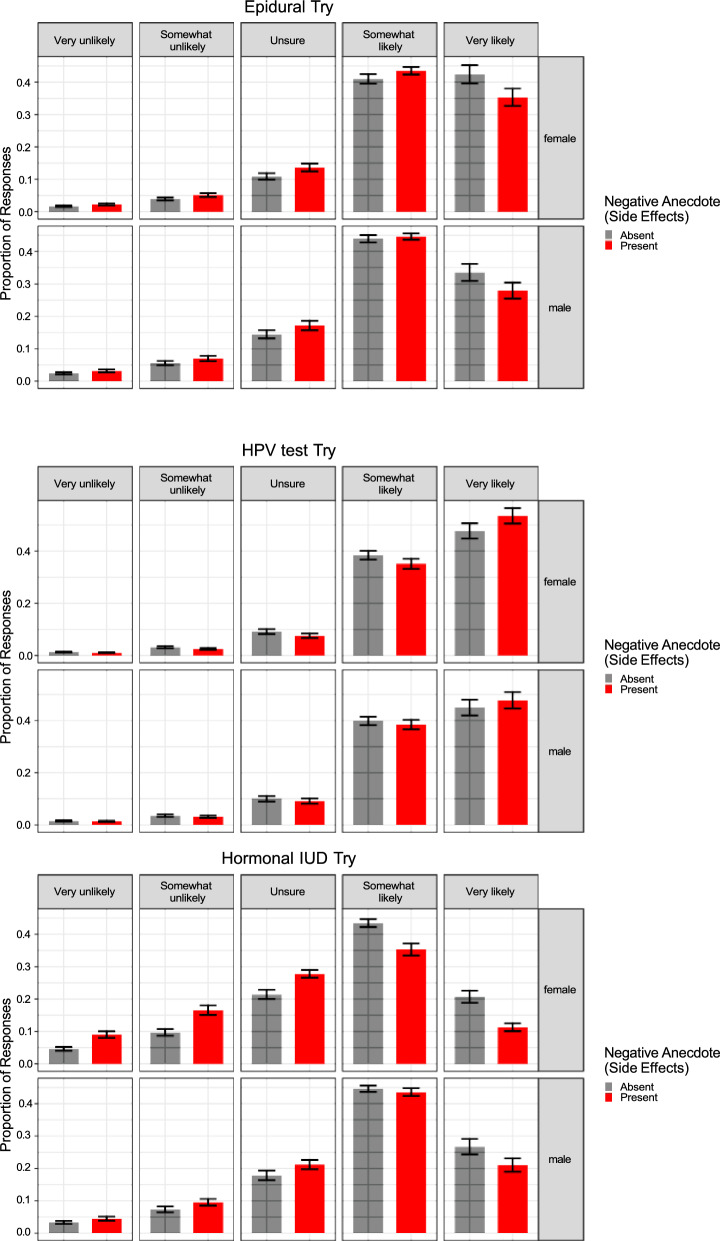
Participant ratings of the likelihood they would try or recommend the treatment across conditions in Experiment 2. Higher Likert scale choices indicate more favorable attitudes toward health topics. The figure indicates that participants presented with negative anecdotal evidence had less favorable attitudes toward treatments after the intervention relative to participants who were not presented with negative anecdotal evidence. The error bars represent standard errors of model-estimated means. The negative anecdotes are labeled as having side effects in order to differentiate between the anecdotes used in Experiments 1a–1c vs. Experiment 2

We first tested whether the inclusion of a negative anecdote would affect participants’ judgments as we saw across Experiments 1a–1c. We fit a Bayesian multivariate cumulative model regressing efficacy attitudes on the presence of an Anecdote (Reference = No Anecdote) and Sex (Reference = Female) and the interaction between these predictors. The summary of this model is shown below. Figures 6 and 7 show the effect of anecdotes across sex and each medical treatment. As predicted, the anecdote affected females’ beliefs about the efficacy of the treatment, along with their willingness to try it, whereas males were only nominally, and weakly, affected by the presence of an anecdote (see Table 3).

**Table 3:** Bayesian mixed–effects cumulative regression predicting efficacy attitudes (Higher = More positive attitudes toward a medical treatment) on Negative Anecdote (0 = No Anecdote), Sex (0 = Female), and the interaction of these predictors

Parameter	Estimate	Error	Lower	Upper
Negative Anecdote (Effective)	− 0.31	0.12	− 0.54	− 0.07
Sex (Effective)	− 0.26	0.13	− 0.51	− 0.01
Anecdote × Sex (Effective)	0.30	0.18	− 0.04	0.63
Negative Anecdote (Try)	− 0.27	0.10	− 0.47	− 0.06
Sex (Try)	− 0.06	0.11	− 0.28	0.15
Anecdote × Sex (Try)	0.11	0.15	− 0.19	0.41

The No Anecdote condition and sex (Female) were the reference groups in this analysis. Lower and Upper indicate the 95% credible intervals for each estimate. Threshold and group- level parameters are omitted for brevity

Note, however, that Sex and Negative Anecdote did not interact. Marginalizing over Sex, we replicated the effects of Experiments 1a–1c, showing that a negative anecdote affected participants’ integration of statistical information, *b*_Effective_ = − 0.18, 95% CI [− 0.35, 0.00]; *b*_Try_ = − 0.22, 95% CI [− 0.37, − 0.06]. Focusing on Fig. 7, we also observed that the effect of negative anecdotes was most pronounced for two of the three treatments—Epidurals and Hormonal IUDs. We consider some possible explanations for finding in the discussion section, but this finding highlights boundary conditions on the effects we observed in Experiment 2.

Contrary to the results of Experiment 1c, icon arrays appeared to have little effect on participants’ attitudes. Although this finding diverges from prior literature (and Experiment 1c), participants in our study received extensive decision aids in which icon arrays played only a small role. In an exploratory analysis, we also fit a model to determine if participants’ age was related to participants’ decisions, but we observed no effect of this predictor. The details of these analyses are located in the Supplement.

## Discussion

People have access to more medical information than ever before. From journal articles to online forums, people must determine what information is relevant and reliable to make medical decisions. People also encounter and seek out health information over social media platforms (Lim et al., 2022), which could lead to uptake of medical misinformation (Suarez-Lledo & Alvarez-Galvez, 2021), including about issues like women’s reproductive health and vaccine safety (Patev & Hood, 2021). How do people make medical decisions under these circumstances?

Across four experiments, we observed that negative anecdotes reduced the perceived effectiveness of, and willingness to try, a variety of medical treatments. We observed this effect despite the fact that either the negative anecdotes added no new information that was not subsumed by the summary statistics participants were presented with (Experiments 1a–1c) or the negative anecdotes did provide new information but were paired with thorough decision aids (Experiment 2). Negative anecdotes affect participants’ decisions even when participants were provided with icon arrays—a data visualization tool specifically designed to aid people’s understanding of complex statistical information.

The information people confront often includes anecdotal information which will rarely be accompanied by data from a clinical trial; people cannot directly compare both pieces of information. Additionally, people are drawn more toward news that is negative than they are to positive news (Robertson et al., 2023). For example, tweets about side effects from the Covid vaccine have gone viral, but positive anecdotes highlighting the success of the vaccine are unlikely to. Thus, not only does negative information have a stronger influence on decision making than positive information, but negative information is more attention grabbing and more likely to go viral. Consequently, our findings are particularly notable because they demonstrate how strong the effect of negative anecdotes on decision making can be. We found that they affected participants decisions even when participants could directly compare them to data from a clinical trial.

What aspects of negative anecdotes produce such consistent effects? There are several components of the anecdotes we used for our studies, and “in the wild,” that could affect people’s responses. First, the anecdotes in Experiment 2 contained language that was more emotional than the anecdotes in Experiments 1a–1c (see Table 2 for an overview of all anecdotes used). While we aimed to control for the emotional intensity by selecting representative anecdotes, an inherent limitation of our design was that these anecdotes were taken from real people describing their experience with reproductive treatments.

Thus, they were not manipulated to equate, remove, or otherwise augment their emotional intensity. On the other hand, the anecdotes people hear in everyday life have these same properties, making this aspect of our design a strength. Additionally, the decision aids and anecdotes in Experiment 2 mention potential side effects, which might raise concerns about the safety of these treatments, along with participants’ evaluation of the treatment’s efficacy. We would expect people to have stronger responses to anecdotes where a patient experienced side effects, although this fact alone cannot account for the findings from Experiments 1a–1c.

The anecdotes that people find online often contain misinformation, which could also exacerbate the effects of negative anecdotes. Misinformation regarding the HPV vaccine is common on social media sites (Suarez-Lledo & Alvarez-Galvez, 2021), and a study that reviewed the most widely viewed YouTube videos about vaccines found that 65% of videos discouraged their use (Basch et al., 2017). Our experiments only included anecdotes with accurate information. In Experiment 2, even when the anecdotes included negative side effects, this information was consistent with the potential side effects listed in the decision aids. Given how quickly misinformation spreads in social networks (Vosoughi et al., 2018), additional research is necessary to identify how misinformation contained in anecdotes impacts medical decisions.

## Limitations and future directions

For some of the treatments described in Experiment 2, such as the Hormonal IUDs, there are alternative treatments available (e.g., birth control pills). It is possible then that people would be less deterred by negative anecdotes when there are no alternative options for a health treatment, or when the trade-off between the perceived consequences of the disease and the side effects of that treatment—its safety—differ (Horne et al., 2015). We focused on how anecdotes impact the perceived efficacy of treatments and people’s willingness to try these treatments. But anecdotal information often relates to the side effects of the treatments, which concerns *safety* rather than efficacy. Although we had participants make decisions about their willingness to try a treatment because we assumed this would include some evaluation of its safety, subsequent investigations could address the impact of anecdotal information on perceived safety more directly.

We also need to consider the potential differences between laboratory experiments and people’s experiences using decision aids in the real world. Participants were required to spend a sustained amount of time reading these decision aids, but patients would likely spend more time and consideration with the decision aids in non-laboratory settings, particularly if they were discussing prospective treatments with a medical professional. Medical personnel might also be available to discuss the decision aid and answer any questions a patient might have. Additionally, while we used existing decision aids from medical institutions, there are many ways to present medical and statistical information and other decision aids might be more effective in relaying this information. In our experiments, some of the icon arrays depicted efficacy (e.g., how many people would get pregnant in a year while on the IUD), whereas others depicted the likelihood of side effects (e.g., how many people who get the epidural will experience severe side effects). These differences could also impact people’s interpretation of the risks and benefits of a treatment. Different decision aids or approaches to presenting decision aids might be more effective in tempering the effects of negative anecdotes.

In Experiment 2, we observed that the effects of a negative anecdote were not as pronounced for the HPV test. HPV tests are not a treatment, rather they are a preventative test that can lead to further treatments (conditional on the results of the test). People’s beliefs and attitudes regarding treatments vs. preventative tests may differ in nuanced ways we’ve not established. For example, attitudes toward cancer screenings are generally positive, especially if patients have a family history of cancer (Yeğenler et al., 2023). Studies have also observed a high rate of women (83%) who get tested for HIV (Fan et al., 2018). The “illusion of certainty,” in which people believe a test outcome is certain, might further contribute to a willingness to participate in preventative *tests* more than preventative *treatments* (Gigerenzer et al., 2007). Women might be more open to getting the HPV test in particular because it can be done at the same time as a routine pap smear (a process known as cotesting; Institute, 2022). Additional research is needed to more directly test the possibility that treatments and preventative tests are differentially affected by negative anecdotes, but Experiment 2 suggests that the effects of anecdotes on people’s decisions were nominally strongest for Hormonal IUDs and Epidurals. A direct and sustained investigation is necessary to resolve these questions.

There are other important qualifications of the effects of negative anecdotes on decision making in the present studies. First, we did not observe that positive anecdotes boosted the perceived credibility of clinical trials, suggesting that anecdotes are particularly effective when they undermine a clinical trial or raise the salience of side effects. Further research is necessary to establish the generalizability of this finding. Second, we did not observe an interaction between sex of the participant and the anecdote for Experiment 2 on reproductive health decisions, although nominally it appears females were more affected by the anecdote than were males. This suggests that the effect of the anecdote may be related to the decision maker’s capacity to think they could have to make this same decision, or empathize with someone who had to make this decision. This possibility is consistent with a recent meta-analysis on anecdotal bias where anecdotes have bigger effects when they concern a decision more relevant to the decision maker (Freling et al., 2020). Thus, it is quite possible that making the anecdote more compelling could make men more empathetic, increasing its effect (Argo et al., 2008). As we noted, we did not manipulate the anecdotes provided in Experiment 2 because our primary aim was conducting a more ecologically valid study of the effect of negative anecdotes on medical decision making. In any case, this exploratory result suggests an important direction for future research—why are anecdotes simply ineffective for some; in this case, roughly half of the participants in Experiment 2.

Along these lines, another question is how our effects would generalize if the treatments primarily concerned males—we focused on women’s reproductive health, in part, because of the lack of psychological research in the domain of women’s healthcare. (We note that HPV testing is available to men in some countries, and thus one of the treatments we investigated may have been viewed as relevant to both men and women.) There are some analogous reproductive treatments for men that further research could address (e.g., STI testing, vasectomies, and drugs for erectile dysfunction). In these cases, we expect anecdotal information would have a stronger effect on men rather than women, an important question for future research.

It is also likely that individual differences affect how people understand and consider statistical and anecdotal information. For example, education might affect people’s reactions to anecdotes about health information; we did not collect these data in our studies because we were interested in average effects in the population. Still, people who have higher levels of education, for example, might rely on the statistical information more heavily than people with lower numeracy or who have lower levels of formal education (Liberali et al., 2012). Other individual differences may also play a role: for instance, people who are loss-averse when making risky decisions (Brooks & Zank, 2005), or people who exhibit stronger negativity biases (Baumeister et al., 2001) might be more sensitive to negative information about a treatment compared to positive information.

The present experiments raise a large number of key questions on the impact of anecdotes on medical decision, but our studies nonetheless highlight their persistent effects. It is striking that a single anecdote can impact reasoning even when presented with medical decision aids or in the presence of data from a clinical trial. Everyday experience suggests that when people research and discuss their medical decisions, they come across not one, but multiple negative anecdotes. In certain cases, people seek these anecdotes out, particularly when they have broad concerns about a prospective treatment (Van Riel et al., 2017). Consequently, we expect that the findings reported here provide a conservative estimate of the effect of anecdotal information on people’s reasoning. Thus, the present research highlights the need for new research aimed at countering the effects of anecdotal information.

### Supplementary Information

Additional file 1. Supplemental Materials.

## Data Availability

We preregistered the data collection plan for all experiments. The raw data and code used to generate our analyses and figures are also available on the Open Science Framework. The final analysis scripts, data, and Supplementary Online Materials (SOM) for all projects can be found at https://osf.io/fcjgd/.
