# Supplementary material for: Anecdotes impact medical decisions even when presented with statistical information or decision aids
Source: Cogn Res Princ Implic. 2024 Aug 26;9:51. doi: 10.1186/s41235-024-00577-3 (PMC11345347; doi:10.1186/s41235-024-00577-3)
Supplement: Supplementary file 1 — Additional file 1. Supplemental Materials. [file 41235_2024_577_MOESM1_ESM.pdf]

Supplemental Materials for:

Anecdotes impact medical decisions even when presented with statistical information or decision-aids

Emily N. Line

Department of Psychology, University of Illinois, Urbana-Champaign

Sara Jaramillo

Department of Psychology, University of Pittsburgh

Micah Goldwater

School of Psychology, The University of Sydney

Zachary Horne

Department of Psychology, University of Edinburgh

Supplemental Materials for:

Anecdotes impact medical decisions even when presented with statistical information or decision-aids

Table S1

The Experimental Vignettes Used in Experiment 1a

Statistics condition
For years, people have struggled with regularly occurring headaches and an inability to focus at work. Many people also describe feeling like they have no energy while they are at work, even when they sleep well the previous night. Doctors hoped to tackle this problem. There are many products on the market that claim to help these symptoms including B12 shots. Doctors were unsure whether to recommend these shots because there had been few clinical controlled studies on the effectiveness of B12 shots. In 2012, a team of well-regarded physicians and clinicians conducted a clinical trial to determine whether B12 shots can help their patients. After a two year trial with 1,000 participants, their study revealed that 87.3% of people who received a B12 injection had fewer headaches and improved cognitive functioning. Those participants in the study also reported having more energy at work. There were few side effects reported by the participants in the study who failed to benefit from the B12 injections. These participants continued to have headaches, lack of energy, and problems focusing.
**Positive Anecdote condition**
Jamie Mitchell is 26 years old single mother. Jamie has one daughter who is three years old. For the past year, Jamie has been struggling with reoccurring headaches, feeling frequently tired, and having a hard time focusing especially at work. Jamie tried changing to a healthier diet, but her symptoms persisted. “It’s usually get up, go to work, come home, make dinner,” she said. “I’m kind of just going through the motions.” Jamie was upset because she was beginning to feel that her symptoms were affecting her relationship with her daughter. She just didn’t have the energy to spend quality time with her after struggling through another day at work. Jamie went to her doctor and expressed her concern. Jamie’s doctor recommended that she participate in a new clinical trial that was examining the effects of B12 on headaches and cognitive functioning. Jamie agreed to participate in the trial over the course of the next year. Jamie received a B12 injection and experienced that she had a reduction in the number of her headaches. She was also more focused and had more energy. Because she had more energy and focus, Jamie was able to play with her daughter and read to her every night.

Statistics + Positive Anecdote condition

For years, people have struggled with regularly occurring headaches and an inability to focus at work. Many people also describe feeling like they have no energy while they are at work, even when they sleep well the previous night. Doctors hoped to tackle this problem. There are many products on the market that claim to help these symptoms including B12 shots. Doctors were unsure whether to recommend these shots because there had been few clinical controlled studies on the effectiveness of B12 shots. A team of well-regarded physicians and clinicians conducted a clinical trial to determine whether B12 shots can help their patients. After a two year trial with 1,000 participants, their study revealed that 87.3% of people who received a B12 injection had fewer headaches and improved cognitive functioning. Participants in the study also reported having more energy at work. There were few side effects reported by the participants in the study who failed to benefit from the B12 injections. These participants continued to have headaches, lack of energy, and problems focusing.

Now you will read about the experience of one of the subjects in the study.

Jamie Mitchell is 26 years old single mother. Jamie has one daughter who is three years old. For the past year, Jamie has been struggling with reoccurring headaches, feeling frequently tired, and having a hard time focusing especially at work. Jamie tried changing to a healthier diet, but her symptoms persisted. “It’s usually get up, go to work, come home, make dinner,” she said. “I’m kind of just going through the motions.” Jamie was upset because she was beginning to feel that her symptoms were affecting her relationship with her daughter. She just didn’t have the energy to spend quality time with her after struggling through another day at work. Jamie went to her doctor and expressed her concern. Jamie’s doctor recommended that she participate in a new clinical trial that was examining the effects of B12 on headaches and cognitive functioning. Jamie agreed to participate in the trial over the course of the next year. Jamie received a B12 injection and experienced that she had a reduction in the number of her headaches. She was also more focused and had more energy. Because she had more energy and focus, Jamie was able to play with her daughter and read to her every night.

Statistics + Negative Anecdote condition

For years, people have struggled with regularly occurring headaches and an inability to focus at work. Many people also describe feeling like they have no energy while they are at work, even when they sleep well the previous night. Doctors hoped to tackle this problem. There are many products on the market that claim to help these symptoms including B12 shots. Doctors were unsure whether to recommend these shots because there had been few clinical controlled studies on the effectiveness of B12 shots. A team of well-regarded physicians and clinicians conducted a clinical trial to determine whether B12 shots can help their patients. After a two year trial with 1,000 participants, their study revealed that 87.3% of people who received a B12 injection had fewer headaches and improved cognitive functioning. Participants in the study also reported having more energy at work. There were few side effects reported by the participants in the study who failed to benefit from the B12 injections. These participants continued to have headaches, lack of energy, and problems focusing.

Now you will read about the experience of one of the subjects in the study.

Jamie Mitchell is 26 years old single mother. Jamie has one daughter who is three years old. For the past year, Jamie has been struggling with reoccurring headaches, feeling frequently tired, and having a hard time focusing especially at work. Jamie tried changing to a healthier diet, but her symptoms persisted. “It’s usually get up, go to work, come home, make dinner,” she said. “I’m kind of just going through the motions.” Jamie was upset because she was beginning to feel that her symptoms were affecting her relationship with her daughter. She just didn’t have the energy to spend quality time with her after struggling through another day at work. Jamie went to her doctor and expressed her concern. Jamie’s doctor recommended that she participate in a new clinical trial that was examining the effects of B12 on headaches and cognitive functioning. Jamie agreed to participate in the trial over the course of the next year. Jamie received a B12 injection and her headaches, lack of energy, and inability to focus persisted. This frustrated Jamie as she was unable to play with her daughter or read to her because of her symptoms.

Table S2

Pretest Questionnaire Used in Experiments 1a-1c.

Are you familiar with people receiving B12 shots as a medical treatment?
Yes; No
Do you or have you received B12 shots as a medical treatment?
Yes; No
Are you considering receiving B12 shots as a medical treatment?
Yes; No
Based on what you know, do you think B12 shots are an effective medical treatment?
Not effective at all; Slightly effective; Moderately effective; Very effective; Extremely effective

Table S3

Posttest Questionnaire Used in Experiments 1a-1c.

How effective do you think B12 shots are as a medical treatment?

Likert scale used in Experiments 1a-1c: *Not effective at all; Slightly effective; Moderately effective; Very effective; Extremely effective*

How likely are you to try B12 shots?

Likert scale used in Experiment 1a: *None at all; A little; A moderate amount; A lot; A great deal*

Likert scale used in Experiments 1b and 1c: *Very unlikely; Unlikely; Somewhat unlikely; Somewhat likely; Likely; Very likely*

How likely are you to give B12 shots to your child (if applicable)?

Likert scale used in Experiment 1a: *None at all; A little; A moderate amount; A lot; A great deal*

Likert scale used in Experiments 1b and 1c: *Very unlikely; Unlikely; Somewhat unlikely; Somewhat likely; Likely; Very likely*

Table S4

The Naturalism Scale Used in Experiment 1a

Are you familiar with people receiving B12 shots as a medical treatment?
Strongly disagree; Disagree; Somewhat disagree; Neither agree nor disagree; Somewhat agree; Agree; Strongly agree
In some cases, it's fair to say that science has improved on nature.
I'm always wary of any chemicals that were developed in a lab.
Overall, advances in chemistry and the creation of synthetic chemicals have done more harm than good or human health.
Childbirth should take place at home rather than in a hospital.
I'm not concerned about preservatives in food.

Table S5

The Medical Skepticism Scale Used in Experiments 1a-1c

Pharmaceutical companies are more interested in making money than in helping people be healthy.<br>**Likert scale used in Experiment 1a:** *Strongly disagree; Disagree; Somewhat disagree; Neither agree nor disagree; Somewhat agree; Agree; Strongly agree*<br>**Likert scale used in Experiments 1b and 1c:** *Strongly disagree; Disagree; Somewhat disagree; Somewhat agree; Agree; Strongly agree*
FDA regulations ensure that approved pharmaceutical drugs are safe.
Pharmaceutical companies put pressure on the FDA and CDC to suppress negative findings.
Most researchers are biased against the idea that medications or vaccinations could cause health problems.
Pharmaceutical companies don't make huge profits from the sale of vaccines.
For researchers developing vaccines, safety is a top priority.

Table S6

The Belief in Holistic Medicine Scale Used in Experiments 1a-1c

The body is essentially self-healing and the task of a health care provider is to assist the healing process.<br>**Likert scale used in Experiment 1a:** *Strongly disagree; Disagree; Somewhat disagree; Neither agree nor disagree; Somewhat agree; Agree; Strongly agree*<br>**Likert scale used in Experiments 1b and 1c:** *Strongly disagree; Disagree; Somewhat disagree; Somewhat agree; Agree; Strongly agree*
Physical and mental health are maintained by an underlying energy or vital force.
A patient's symptoms should be regarded as a manifestation of a general imbalance or dysfunction affecting the whole body.
Health and disease are a reflection of balance between positive life forces and negative life forces.

Table S7

The Experimental Vignettes Used in Experiment 1b

Statistics condition
For years, people have struggled with regularly occurring headaches and an inability to focus at work. Many people also describe feeling like they have no energy while they are at work, even when they sleep well the previous night. Doctors hoped to tackle this problem. There are many products on the market that claim to help these symptoms including B12 shots. Doctors were unsure whether to recommend these shots because there had been few clinical controlled studies on the effectiveness of B12 shots. A team of well-regarded physicians and clinicians conducted a clinical trial to determine whether B12 shots can help their patients. After a two year trial with 1,000 participants, their study revealed that B12 injections worked for 87.3% of participants and failed to work for 12.7%. The participants that the treatment did work for had fewer headaches and improved cognitive functioning. These participants also reported having more energy at work. There were few side effects reported by the participants in the study who failed to benefit from the B12 injections. These participants continued to have headaches, lack of energy, and problems focusing.
Or
For years, people have struggled with regularly occurring headaches and an inability to focus at work. Many people also describe feeling like they have no energy while they are at work, even when they sleep well the previous night. Doctors hoped to tackle this problem. There are many products on the market that claim to help these symptoms including B12 shots. Doctors were unsure whether to recommend these shots because there had been few clinical controlled studies on the effectiveness of B12 shots. A team of well-regarded physicians and clinicians conducted a clinical trial to determine whether B12 shots can help their patients. After a two year trial with 1,000 participants, their study revealed that B12 injections failed to work for 12.7% of participants and worked for 87.3%. The participants that the treatment did work for had fewer headaches and improved cognitive functioning. These participants also reported having more energy at work. There were few side effects reported by the participants in the study who failed to benefit from the B12 injections. These participants continued to have headaches, lack of energy, and problems focusing.

Statistics + Positive Anecdote condition
*This vignette is the same as presented in Experiment 1 (see Table 1). However, a new positive anecdote was included in the anecdote portion of the script. We randomized which participants in the Statistics + Positive Anecdote condition would receive the original anecdote or the new positive anecdote.
John Irving is a 35 year old construction worker and owns a dog named Max. For the past 2 years, John has frequently felt tired at work and has a hard time focusing. In the past six months, these symptoms seem to have gotten worse and are coupled with painful headaches. John is worried and doesn't want these symptoms to result in an accident at work. "For a long time, I thought my low energy was because of the heavy physical demands of my job. I cut out fast food and started getting more sleep, but I'd still wake up feeling like I hadn't slept." John went to his doctor and expressed his concern. John's doctor recommended that he participate in a new clinical trial that was examining the effects of B12 on headaches and cognitive functioning. John agreed to participate in the trial over the course of the next year. John received a B12 injection and experienced that he had more energy and focus at work. Because of this, he was able to take his dog Max on walks more frequently. John also noticed that the headaches that he had been getting had gone away.

Statistics + Negative Anecdote condition
*This vignette is the same as presented in Experiment 1 (see Table 1). However, a new negative anecdote was included in the anecdote portion of the script. We randomized which participants in the Statistics + Negative Anecdote condition would receive the original anecdote or the new negative anecdote.
John Irving is a 35 year old construction worker and owns a dog named Max. For the past 2 years, John has frequently felt tired at work and has a hard time focusing. In the past six months, these symptoms seem to have gotten worse and are coupled with painful headaches. John is worried and doesn't want these symptoms to result in an accident at work. "For a long time, I thought my low energy was because of the heavy physical demands of my job. I cut out fast food and started getting more sleep, but I'd still wake up feeling like I hadn't slept." John went to his doctor and expressed his concern. John's doctor recommended that he participate in a new clinical trial that was examining the effects of B12 on headaches and cognitive functioning. John agreed to participate in the trial over the course of the next year. John received a B12 injection and his headaches, lack of energy, and inability to focus persisted. This frustrated John as he continued to struggle at work and doesn't have the energy to take his dog Max on walks.

Statistics + Positive and Negative Anecdote condition
*This condition combined anecdotes from the Statistics + Positive Anecdote condition and the Statistics + Negative Anecdote condition. After reading the first anecdote, participants were told “Now you’ll read about another person’s experience in the study".

Table S8

The Experimental Vignettes Used in Experiment 1c

Statistics condition
*This vignette is the same as presented in Experiment 1a (see Table S1).

Statistics + Icon Array condition
*This vignette is the same as presented in Experiment 1a (see Table S1). However now participants are presented with an image and accompanying text. (See Figure 1 below.)

Statistics + Negative Anecdote condition
*This vignette is the same as presented in Experiment 1a (see Table S1).

Statistics + Icon and Negative Anecdote condition
*This vignette is the same as presented in Experiment 1a (see Table S1). However now participants are presented with two images and accompanying text. (See Figures 1 and 2 below).

Experiment 1a Supplemental Results

In addition to our main model which regressed B-12 beliefs on Condition (Reference = Statistics condition) and pretest beliefs about the efficacy of B-12, we conducted a subsequent exploratory analysis to determine if pretest beliefs interacted with condition. To evaluate out-of-sample model fit, we ran 10-fold cross-validation on each model. These analyses showed that the exploratory model did not improve model fit over our original model. The difference in elpd (using the main model as the reference) was -1.9, SE = 4.1. The full results of the exploratory model are available on OSF.

Experiment 1c Supplemental Results

We conducted several exploratory analyses to determine if participant characteristics interacted with the anecdote and icon array conditions. We regressed B-12 beliefs on Condition (Reference = Statistics condition), Icon array (Reference = no array), and Gender (Reference = Male), and their interactions, while controlling for pretest beliefs about the efficacy of B-12. There were no meaningful interactions involving Gender for beliefs about efficacy, trying the treatment, or giving the treatment to their child. There were also no meaningful main effects of Gender for these measures. See Table S9 for the full results. We also regressed B-12 beliefs on Condition (Reference = Statistics condition), Icon array (Reference = no array), and Age (standardized), and their interactions, while controlling for pretest beliefs about the efficacy of B-12. There were no meaningful interactions involving Age for beliefs about efficacy, trying the treatment, or giving the treatment to their child. There were also no meaningful main effects of Age for these measures. See Table S10 for the full results.

We collected measures for two different scales related to participants' beliefs about health treatments. The first scale was the Belief in Holistic Medicine Scale (see Table S6), which contains items regarding beliefs about holistic practices. We regressed B-12 beliefs on Condition (Reference = Statistics condition), Icon array (Reference = no array), and Holistic Beliefs, and their interactions, while controlling for pretest beliefs about the efficacy of B-12. There were no meaningful interactions or main effects involving Holistic Beliefs. See Table S11 for the full results.

The second scale was the Medical Skepticism Scale (also referred to as Pharma in the data), which assesses people's beliefs about pharmaceutical practices and medicine (see Table S5). We regressed B-12 beliefs on Condition (Reference = Statistics condition), Icon array (Reference = no array), and Medical Skepticism, and their interactions, while controlling for pretest beliefs about the efficacy of B-12. There was a main effect of Medical Skepticism for giving a B-12 treatment to a child, $b_{Child} = -0.45, 95\% CI = [-0.72, -0.18]$, in that people who are more skeptical of pharmaceutical companies are less likely to give B-12 to their

children. However, this effect was only present for the child dependent variable, it did not apply to the effectiveness and trying the treatment for oneself measures. See Table S12 for the full results.

Table S13

The Experimental Vignettes Used in Experiment 2

Epidural - Statistics condition
Imagine you or a loved one is pregnant and considering receiving an epidural to relieve birthing pain. You decide to go online to learn more information about epidurals.
Now we will ask you to read some information about epidurals.
Pregnant patients have a variety of options for birth pain relief. One of these options is an **epidural**.
**Pregnancy: Should I have an epidural during childbirth?**
You may want to have a say in this decision, or you may simply want to follow your doctor's recommendation. Either way, this information will help you understand what your choices are so that you can talk to your doctor about them.
**Your options**
Have an epidural to control pain during childbirth. Don't have an epidural. Instead, use other methods to control the pain.
**Key points to remember**
An epidural is medicine that numbs your lower body so that childbirth doesn't hurt as much. The dose can be changed to make you partly numb or completely numb, depending on how much pain you're feeling.
For most women, this is a personal decision that depends on two things: how worried you are about having pain and how important natural childbirth (labor without pain medicine) is to you. An epidural is considered the most effective and easily adjustable type of pain relief for childbirth.
Epidurals are very common. But there are some risks and possible side effects you should know about.
Labor pain in unpredictable. You may have more pain than you expected. You may plan to have a natural childbirth and then decide you need pain medicine.
**What is an epidural?** An epidural is pain medicine that you get through a very thin tube (catheter) inserted into your back. Your lower body becomes partly or totally numb, depending on how much medicine is used. But you stay awake and alert.
Some hospitals and birthing centers offer a “light epidural” or “walking epidural”. This is a light dose of medicine that makes it possible for you to walk around and to push during contractions. Ask your doctor if your hospital or center offers a “light epidural.”
**Sometimes you can't get an epidural:**
Your labor may happen so fast that there isn't time for an epidural.
You may be in a smaller hospital that doesn't offer them. If you think you may want an epidural, find out ahead of time if they are offered at the hospital or birthing center where you're planning to go.
You may have a health problem that means you can't have an epidural.

Epidural - Statistics condition continued

What are the benefits of having an epidural?

Epidurals are considered the most effective and easily adjustable type of pain relief for childbirth.

After an epidural is started, you can quickly get pain relief if and when you need it during labor and delivery.

The medicine in an epidural doesn't make you sleepy, so you are awake and alert for the delivery.

If you were to end up needing a C-section, the epidural could quickly numb the area below your waist for the surgery.

What are the risks of having an epidural?

Drop in blood pressure. This can lower your baby's heart rate. To help prevent this, you receive fluids through an IV beforehand and are encouraged to lie on your side, which improves blood flow to the baby.

Being unable to feel your contractions and to push. This increases your risk of needing an assisted delivery (forceps or vacuum).

Seizure. This is very rare.

[See Figure 3 for the corresponding image presented to participants.]

What are the side effects?

After delivery with an epidural, you may have:

Back soreness at the catheter site. This isn't common. Some women fear that an epidural causes long-term back pain. But studies have not shown a connection between new back pain and epidural use.

A severe, prolonged headache. This can happen when the spinal cord sheath has accidentally been punctured during the epidural.

In a clinical trial studying the efficacy of epidurals for birth pain relief, the puncture happened to about 1 out of 100 women. That means the puncture does **not** happen to 99 out of 100 women. After the puncture is fixed with a different medicine, the headache usually goes away.

What other methods are used to control labor pain?

Pain medicines. You can get a shot of pain medicine or get it through an IV. The most common medicines used are opioids, also known as narcotics. These medicines:

Help you relax between contractions.

Decrease the pain (but they don't take it away completely).

Have side effects, including drowsiness, nausea, and vomiting.

Are less likely than an epidural to cause you to have a forceps or vacuum delivery.

Epidural - Statistics condition continued

Natural methods. There are also several ways to control pain without using medicine. They include:

Distraction. Walk, play cards, watch TV, take a shower, or read to help take your mind off your contractions.

Massage. Massage of the shoulders and lower back during contractions may ease your pain.

Imagery. For instance, think of contractions as waves rolling over you. Picture a peaceful place, such as a beach or mountain stream, to help you relax between contractions.

Focused breathing. Breathing in a rhythm can distract you from pain. Childbirth education classes teach you different methods of focused breathing.

Nitrous oxide. You can give yourself nitrous oxide (a gas) through a mask when you need pain relief. Nitrous oxide is not available in many places.

Why might your doctor recommend an epidural?

This is usually a personal decision, but an epidural might be recommended in certain situations, such as when:

Your labor pain is so intense that you feel exhausted or out of control. An epidural can help you rest and get focused.

You have a higher than average chance of needing a C-section. If you do need surgery, the epidural would already be in place and you could be quickly numbed.

Compare your options

[See Figure 4 for corresponding table.]

Epidural - Statistics + Icon Array

Imagine you or a loved one is pregnant and considering receiving an epidural to relieve birthing pain. You decide to go online to learn more information about epidurals.

Now we will ask you to read some information about epidurals.

Pregnant patients have a variety of options for birth pain relief. One of these options is an **epidural**.

Pregnancy: Should I have an epidural during childbirth?

You may want to have a say in this decision, or you may simply want to follow your doctor's recommendation. Either way, this information will help you understand what your choices are so that you can talk to your doctor about them.

Your options

Have an epidural to control pain during childbirth. Don't have an epidural. Instead, use other methods to control the pain.

Epidural - Statistics + Icon Array condition continued

Key points to remember

An epidural is medicine that numbs your lower body so that childbirth doesn't hurt as much. The dose can be changed to make you partly numb or completely numb, depending on how much pain you're feeling.

For most women, this is a personal decision that depends on two things: how worried you are about having pain and how important natural childbirth (labor without pain medicine) is to you. An epidural is considered the most effective and easily adjustable type of pain relief for childbirth.

Epidurals are very common. But there are some risks and possible side effects you should know about.

Labor pain in unpredictable. You may have more pain than you expected. You may plan to have a natural childbirth and then decide you need pain medicine.

What is an epidural? An epidural is pain medicine that you get through a very thin tube (catheter) inserted into your back. Your lower body becomes partly or totally numb, depending on how much medicine is used. But you stay awake and alert.

Some hospitals and birthing centers offer a "light epidural" or "walking epidural". This is a light dose of medicine that makes it possible for you to walk around and to push during contractions. Ask your doctor if your hospital or center offers a "light epidural."

Sometimes you can't get an epidural:

Your labor may happen so fast that there isn't time for an epidural.

You may be in a smaller hospital that doesn't offer them. If you think you may want an epidural, find out ahead of time if they are offered at the hospital or birthing center where you're planning to go.

You may have a health problem that means you can't have an epidural.

What are the benefits of having an epidural?

Epidurals are considered the most effective and easily adjustable type of pain relief for childbirth.

After an epidural is started, you can quickly get pain relief if and when you need it during labor and delivery.

The medicine in an epidural doesn't make you sleepy, so you are awake and alert for the delivery.

If you were to end up needing a C-section, the epidural could quickly numb the area below your waist for the surgery.

What are the risks of having an epidural?

Drop in blood pressure. This can lower your baby's heart rate. To help prevent this, you receive fluids through an IV beforehand and are encouraged to lie on your side, which improves blood flow to the baby.

Being unable to feel your contractions and to push. This increases your risk of needing an assisted delivery (forceps or vacuum).

Seizure. This is very rare.

[See Figure 3 for the corresponding image presented to participants.]

Epidural - Statistics + Icon Array condition continued

What are the side effects?

After delivery with an epidural, you may have:

Back soreness at the catheter site. This isn't common. Some women fear that an epidural causes long-term back pain. But studies have not shown a connection between new back pain and epidural use.

A severe, prolonged headache. This can happen when the spinal cord sheath has accidentally been punctured during the epidural.

In a clinical trial studying the efficacy of epidurals for birth pain relief, the puncture happened to about 1 out of 100 women. That means the puncture does **not** happen to 99 out of 100 women. After the puncture is fixed with a different medicine, the headache usually goes away.

Imagine 100 people like you sitting in a cinema. Now, imagine they all choose to receive an epidural to relieve birthing pain. Out of those 100 people, 1 woman will experience a severe headache as the results of spinal cord puncturing.

[See Figure 4 for the corresponding Icon Array.]

What other methods are used to control labor pain?

Pain medicines. You can get a shot of pain medicine or get it through an IV. The most common medicines used are opioids, also known as narcotics. These medicines:

Help you relax between contractions.

Decrease the pain (but they don't take it away completely).

Have side effects, including drowsiness, nausea, and vomiting.

Are less likely than an epidural to cause you to have a forceps or vacuum delivery.

Natural methods. There are also several ways to control pain without using medicine. They include:

Distraction. Walk, play cards, watch TV, take a shower, or read to help take your mind off your contractions.

Massage. Massage of the shoulders and lower back during contractions may ease your pain.

Imagery. For instance, think of contractions as waves rolling over you. Picture a peaceful place, such as a beach or mountain stream, to help you relax between contractions.

Focused breathing. Breathing in a rhythm can distract you from pain. Childbirth education classes teach you different methods of focused breathing.

Nitrous oxide. You can give yourself nitrous oxide (a gas) through a mask when you need pain relief. Nitrous oxide is not available in many places.

Epidural - Statistics + Icon Array condition continued

Why might your doctor recommend an epidural?

This is usually a personal decision, but an epidural might be recommended in certain situations, such as when:

Your labor pain is so intense that you feel exhausted or out of control. An epidural can help you rest and get focused.

You have a higher than average chance of needing a C-section. If you do need surgery, the epidural would already be in place and you could be quickly numbed.

Compare your options

[See Figure 4 for corresponding table.]

Epidural - Negative Anecdote condition

Imagine you or a loved one is pregnant and considering receiving an epidural to relieve birthing pain. You decide to go online to learn more information about epidurals.

Now you'll read about Jessica's experience receiving an epidural.

"I didn't really think too much about how I was going to handle labor pain. When I was in the middle of labor, they told me I could have an epidural, and I just said yes. I didn't like it at all. I couldn't feel enough to push. Then, I had a bad headache for days afterwards. Of course, it only matters that my baby is healthy, but I won't have an epidural again."

Now you'll read some information about epidurals.

Pregnant patients have a variety of options for birth pain relief. One of these options is an **epidural**.

Pregnancy: Should I have an epidural during childbirth?

You may want to have a say in this decision, or you may simply want to follow your doctor's recommendation. Either way, this information will help you understand what your choices are so that you can talk to your doctor about them.

Key points to remember

An epidural is medicine that numbs your lower body so that childbirth doesn't hurt as much. The dose can be changed to make you partly numb or completely numb, depending on how much pain you're feeling.

For most women, this is a personal decision that depends on two things: how worried you are about having pain and how important natural childbirth (labor without pain medicine) is to you. An epidural is considered the most effective and easily adjustable type of pain relief for childbirth.

Epidurals are very common. But there are some risks and possible side effects you should know about.

Labor pain in unpredictable. You may have more pain than you expected. You may plan to have a natural childbirth and then decide you need pain medicine.

Epidural - Negative Anecdote condition continued

What is an epidural? An epidural is pain medicine that you get through a very thin tube (catheter) inserted into your back. Your lower body becomes partly or totally numb, depending on how much medicine is used. But you stay awake and alert.

Some hospitals and birthing centers offer a "light epidural" or "walking epidural". This is a light dose of medicine that makes it possible for you to walk around and to push during contractions. Ask your doctor if your hospital or center offers a "light epidural."

Sometimes you can't get an epidural:

Your labor may happen so fast that there isn't time for an epidural.

You may be in a smaller hospital that doesn't offer them. If you think you may want an epidural, find out ahead of time if they are offered at the hospital or birthing center where you're planning to go.

You may have a health problem that means you can't have an epidural.

What are the benefits of having an epidural?

Epidurals are considered the most effective and easily adjustable type of pain relief for childbirth.

After an epidural is started, you can quickly get pain relief if and when you need it during labor and delivery.

The medicine in an epidural doesn't make you sleepy, so you are awake and alert for the delivery.

If you were to end up needing a C-section, the epidural could quickly numb the area below your waist for the surgery.

What are the risks of having an epidural?

Drop in blood pressure. This can lower your baby's heart rate. To help prevent this, you receive fluids through an IV beforehand and are encouraged to lie on your side, which improves blood flow to the baby.

Being unable to feel your contractions and to push. This increases your risk of needing an assisted delivery (forceps or vacuum).

Seizure. This is very rare.

What are the side effects?

After delivery with an epidural, you may have:

Back soreness at the catheter site. This isn't common. Some women fear that an epidural causes long-term back pain. But studies have not shown a connection between new back pain and epidural use.

A severe, prolonged headache. This can happen when the spinal cord sheath has accidentally been punctured during the epidural.

In a clinical trial studying the efficacy of epidurals for birth pain relief, the puncture happened to about 1 out of 100 women. That means the puncture does **not** happen to 99 out of 100 women. After the puncture is fixed with a different medicine, the headache usually goes away.

Jessica, the patient you read about who received an epidural, was a participant in this clinical trial.

Epidural - Negative Anecdote condition continued

What other methods are used to control labor pain?

Pain medicines. You can get a shot of pain medicine or get it through an IV. The most common medicines used are opioids, also known as narcotics. These medicines:

Help you relax between contractions.

Decrease the pain (but they don't take it away completely).

Have side effects, including drowsiness, nausea, and vomiting.

Are less likely than an epidural to cause you to have a forceps or vacuum delivery.

Natural methods. There are also several ways to control pain without using medicine. They include:

Distraction. Walk, play cards, watch TV, take a shower, or read to help take your mind off your contractions.

Massage. Massage of the shoulders and lower back during contractions may ease your pain.

Imagery. For instance, think of contractions as waves rolling over you. Picture a peaceful place, such as a beach or mountain stream, to help you relax between contractions.

Focused breathing. Breathing in a rhythm can distract you from pain. Childbirth education classes teach you different methods of focused breathing.

Nitrous oxide. You can give yourself nitrous oxide (a gas) through a mask when you need pain relief. Nitrous oxide is not available in many places.

Why might your doctor recommend an epidural?

This is usually a personal decision, but an epidural might be recommended in certain situations, such as when:

Your labor pain is so intense that you feel exhausted or out of control. An epidural can help you rest and get focused.

You have a higher than average chance of needing a C-section. If you do need surgery, the epidural would already be in place and you could be quickly numbed.

Compare your options

[See Figure 4 for corresponding table.]

Epidural - Negative Anecdote condition

Imagine you or a loved one is pregnant and considering receiving an epidural to relieve birthing pain. You decide to go online to learn more information about epidurals.

Now you'll read about Jessica's experience receiving an epidural.

"I didn't really think too much about how I was going to handle labor pain. When I was in the middle of labor, they told me I could have an epidural, and I just said yes. I didn't like it at all. I couldn't feel enough to push. Then, I had a bad headache for days afterwards. Of course, it only matters that my baby is healthy, but I won't have an epidural again."

Now you'll read some information about epidurals.

Pregnant patients have a variety of options for birth pain relief. One of these options is an **epidural**.

Epidural - Negative Anecdote + Icon Array condition

Pregnancy: Should I have an epidural during childbirth?

You may want to have a say in this decision, or you may simply want to follow your doctor's recommendation. Either way, this information will help you understand what your choices are so that you can talk to your doctor about them.

Key points to remember

An epidural is medicine that numbs your lower body so that childbirth doesn't hurt as much. The dose can be changed to make you partly numb or completely numb, depending on how much pain you're feeling.

For most women, this is a personal decision that depends on two things: how worried you are about having pain and how important natural childbirth (labor without pain medicine) is to you. An epidural is considered the most effective and easily adjustable type of pain relief for childbirth.

Epidurals are very common. But there are some risks and possible side effects you should know about.

Labor pain in unpredictable. You may have more pain than you expected. You may plan to have a natural childbirth and then decide you need pain medicine.

What is an epidural? An epidural is pain medicine that you get through a very thin tube (catheter) inserted into your back. Your lower body becomes partly or totally numb, depending on how much medicine is used. But you stay awake and alert.

Some hospitals and birthing centers offer a "light epidural" or "walking epidural". This is a light dose of medicine that makes it possible for you to walk around and to push during contractions. Ask your doctor if your hospital or center offers a "light epidural."

Sometimes you can't get an epidural:

Your labor may happen so fast that there isn't time for an epidural.

You may be in a smaller hospital that doesn't offer them. If you think you may want an epidural, find out ahead of time if they are offered at the hospital or birthing center where you're planning to go.

You may have a health problem that means you can't have an epidural.

What are the benefits of having an epidural?

Epidurals are considered the most effective and easily adjustable type of pain relief for childbirth.

After an epidural is started, you can quickly get pain relief if and when you need it during labor and delivery.

The medicine in an epidural doesn't make you sleepy, so you are awake and alert for the delivery.

If you were to end up needing a C-section, the epidural could quickly numb the area below your waist for the surgery.

Epidural - Negative Anecdote + Icon Array condition continued

What are the risks of having an epidural?

Drop in blood pressure. This can lower your baby's heart rate. To help prevent this, you receive fluids through an IV beforehand and are encouraged to lie on your side, which improves blood flow to the baby.

Being unable to feel your contractions and to push. This increases your risk of needing an assisted delivery (forceps or vacuum).

Seizure. This is very rare.

[See Figure 3 for the corresponding image presented to participants.]

What are the side effects?

After delivery with an epidural, you may have:

Back soreness at the catheter site. This isn't common. Some women fear that an epidural causes long-term back pain. But studies have not shown a connection between new back pain and epidural use.

A severe, prolonged headache. This can happen when the spinal cord sheath has accidentally been punctured during the epidural.

In a clinical trial studying the efficacy of epidurals for birth pain relief, the puncture happened to about 1 out of 100 women. That means the puncture does **not** happen to 99 out of 100 women. After the puncture is fixed with a different medicine, the headache usually goes away.

Jessica, the patient you read about who received an epidural, was a participant in this clinical trial.

Imagine 100 people like you sitting in a cinema. Now, imagine they all choose to receive an epidural to relieve birthing pain. Out of those 100 people, 1 woman will experience a severe headache as the results of spinal cord puncturing.

[See Figure 4 for the corresponding Icon Array.]

What other methods are used to control labor pain?

Pain medicines. You can get a shot of pain medicine or get it through an IV. The most common medicines used are opioids, also known as narcotics. These medicines:

Help you relax between contractions.

Decrease the pain (but they don't take it away completely).

Have side effects, including drowsiness, nausea, and vomiting.

Are less likely than an epidural to cause you to have a forceps or vacuum delivery.

Natural methods. There are also several ways to control pain without using medicine. They include:

Distraction. Walk, play cards, watch TV, take a shower, or read to help take your mind off your contractions.

Massage. Massage of the shoulders and lower back during contractions may ease your pain.

Epidural - Negative Anecdote + Icon Array condition continued

Imagery. For instance, think of contractions as waves rolling over you. Picture a peaceful place, such as a beach or mountain stream, to help you relax between contractions.

Focused breathing. Breathing in a rhythm can distract you from pain. Childbirth education classes teach you different methods of focused breathing.

Nitrous oxide. You can give yourself nitrous oxide (a gas) through a mask when you need pain relief. Nitrous oxide is not available in many places.

Why might your doctor recommend an epidural?

This is usually a personal decision, but an epidural might be recommended in certain situations, such as when:

Your labor pain is so intense that you feel exhausted or out of control. An epidural can help you rest and get focused.

You have a higher than average chance of needing a C-section. If you do need surgery, the epidural would already be in place and you could be quickly numbed.

Compare your options

[See Figure 4 for corresponding table.]

HPV - Statistics condition

Imagine you or a loved one is considering receiving the HPV test. You decide to go online to learn more information about the HPV test.

Now we will ask you to read some information about the HPV test.

A new test is available for patients with a mildly abnormal Pap smear. It is called the **HPV (Human papillomavirus) test**.

Patients can choose whether they would like the new test or the standard usual care for those with a mildly abnormal result, a repeat Pap smear in six months. Both managements are highly effective, however, each has its own advantages and disadvantages.

What is a Pap smear?

The Pap smear checks for changes in the cells at the neck of the womb (the cervix) at the top of the vagina.

It detects early changes in the cells which if left untreated may become cancer.

Most cancers of the cervix take up to 10 years to develop.

When a Pap smear is performed, a small sample of cells is taken from the cervix and is examined under a microscope.

A mildly abnormal Pap result means that very slight changes in the cells have been found. The cause of the cell changes is often not clear. They are commonly caused by the presence of **HPV**.

[See Figure 6 for corresponding image shown to participants.]

HPV - Statistics condition continued

What is HPV?

HPV is one of the most common viral infections. It is thought that over 70% of women will have HPV at some stage during their lifetime.

Recent studies have shown that most cases of cervical cancer are caused by HPV.

The abnormal cells changes **ARE NOT** cancer.

It is important to remember that medical care for abnormal cells of the cervix is **very effective**.

Options for follow-up examination:

HPV test A clinical study was run to study the efficacy of HPV tests. In patients whose Pap smears tested positive for mild abnormalities:

Between 50 and 80 patients in every 100 will test positive for HPV and will be advised to have a colposcopy and biopsy.

Between 20 and 50 patients in every 100 will test negative for HPV and need no further checks for 12 months.

Usual care (repeat Pap testing)

If a patient decides to follow usual care, the chance of having a colposcopy is lower. Between 20 and 40 out of every 100 patients who have a mildly abnormal Pap smear will be advised to have a colposcopy over a two year period. This is because many minor abnormalities clear up by themselves over time.

What is a colposcopy?

A colposcopy is a more detailed examination of the cervix using an instrument called a colposcope, which is like a pair of binoculars on a stand.

The doctor will insert a speculum into the vagina to hold it open, as was done for the Pap smear.

The cervix is painted with a liquid which makes any abnormal areas change color.

The doctor looks through the colposcope to examine the cervix, to see the location and pattern of the abnormal areas.

During the colposcopy, a small sample of tissue, called a **biopsy**, may be taken from any abnormal looking areas of the cervix to confirm a diagnosis.

Timing

HPV testing gives patients the chance of having an earlier test result because they can arrange to have the test straight away. This means that:

If a more serious problem is detected any treatment can be carried out sooner.

Patients have a shorter period to wait for the test results. For some patients this means they experience less anxiety and worry.

In comparison, usual care can sometimes be a long process. Patients have to wait six months for their first follow up Pap smear and it takes over two years until they return to routine screening. This period of time can cause some patients to feel worried for a long time.

HPV - Statistics + Icon Array condition

Imagine you or a loved one is considering receiving the HPV test. You decide to go online to learn more information about the HPV test.

Now we will ask you to read some information about the HPV test.

A new test is available for patients with a mildly abnormal Pap smear. It is called the **HPV (Human papillomavirus) test**.

Patients can choose whether they would like the new test or the standard usual care for those with a mildly abnormal result, a repeat Pap smear in six months. Both managements are highly effective, however, each has its own advantages and disadvantages.

What is a Pap smear?

The Pap smear checks for changes in the cells at the neck of the womb (the cervix) at the top of the vagina.

It detects early changes in the cells which if left untreated may become cancer.

Most cancers of the cervix take up to 10 years to develop.

When a Pap smear is performed, a small sample of cells is taken from the cervix and is examined under a microscope.

A mildly abnormal Pap result means that very slight changes in the cells have been found. The cause of the cell changes is often not clear. They are commonly caused by the presence of **HPV**.

[See Figure 6 for corresponding image shown to participants.]

What is HPV?

HPV is one of the most common viral infections. It is thought that over 70% of women will have HPV at some stage during their lifetime.

Recent studies have shown that most cases of cervical cancer are caused by HPV.

The abnormal cells changes **ARE NOT** cancer.

It is important to remember that medical care for abnormal cells of the cervix is **very effective**.

Options for follow-up examination:

HPV test A clinical study was run to study the efficacy of HPV tests. In patients whose Pap smears tested positive for mild abnormalities:

Between 50 and 80 patients in every 100 will test positive for HPV and will be advised to have a colposcopy and biopsy.

Between 20 and 50 patients in every 100 will test negative for HPV and need no further checks for 12 months.

Usual care (repeat Pap testing)

If a patient decides to follow usual care, the chance of having a colposcopy is lower. Between 20 and 40 out of every 100 patients who have a mildly abnormal Pap smear will be advised to have a colposcopy over a two year period. This is because many minor abnormalities clear up by themselves over time.

HPV - Statistics + Icon Array condition

What is a colposcopy?

A colposcopy is a more detailed examination of the cervix using an instrument called a colposcope, which is like a pair of binoculars on a stand.

The doctor will insert a speculum into the vagina to hold it open, as was done for the Pap smear.

The cervix is painted with a liquid which makes any abnormal areas change color.

The doctor looks through the colposcope to examine the cervix, to see the location and pattern of the abnormal areas.

During the colposcopy, a small sample of tissue, called a **biopsy**, may be taken from any abnormal looking areas of the cervix to confirm a diagnosis.

Imagine 100 people like you sitting in a cinema. Now, imagine they all choose the usual care, a repeated Pap test.

Out of those 100 people, 20-40 will be recommended to have a colposcopy examination over a two year period.

Now, imagine they all choose the HPV test. Out of those 100 people, 50-80 will be recommended to have a colposcopy.

[See Figure 7 for the icon array.]

Timing

HPV testing gives patients the chance of having an earlier test result because they can arrange to have the test straight away. This means that:

If a more serious problem is detected any treatment can be carried out sooner.

Patients have a shorter period to wait for the test results. For some patients this means they experience less anxiety and worry.

In comparison, usual care can sometimes be a long process. Patients have to wait six months for their first follow up Pap smear and it takes over two years until they return to routine screening. This period of time can cause some patients to feel worried for a long time.

HPV - Negative Anecdote condition

Imagine you or a loved one is considering receiving the HPV test. You decide to go online to learn more information about the HPV test.

Now you'll read about Emily's experience receiving the HPV test.

"I was quite annoyed, couldn't really believe it that it happened to me. And because it was sexually transmitted, and because I haven't had a lot of partners. Yeah wasn't very happy at all really....I phoned the helpline and they reassured me, they really did reassure me so I felt quite happy after talking with them....I would say it [the anxiety] lessened over the year because of the chat I had with the helpline...I thought well if I hadn't taken part in that test I would never have known I had it and I knew I was going to have further tests."

HPV - Negative Anecdote condition continued

Now we will ask you to read some information about the HPV test.

A new test is available for patients with a mildly abnormal Pap smear. It is called the **HPV (Human papillomavirus) test**.

Patients can choose whether they would like the new test or the standard usual care for those with a mildly abnormal result, a repeat Pap smear in six months. Both managements are highly effective, however, each has its own advantages and disadvantages.

What is a Pap smear?

The Pap smear checks for changes in the cells at the neck of the womb (the cervix) at the top of the vagina.

It detects early changes in the cells which if left untreated may become cancer.

Most cancers of the cervix take up to 10 years to develop.

When a Pap smear is performed, a small sample of cells is taken from the cervix and is examined under a microscope.

A mildly abnormal Pap result means that very slight changes in the cells have been found. The cause of the cell changes is often not clear. They are commonly caused by the presence of **HPV**.

[See Figure 6 for corresponding image shown to participants.]

What is HPV?

HPV is one of the most common viral infections. It is thought that over 70% of women will have HPV at some stage during their lifetime.

Recent studies have shown that most cases of cervical cancer are caused by HPV.

The abnormal cells changes **ARE NOT** cancer.

It is important to remember that medical care for abnormal cells of the cervix is **very effective**.

Options for follow-up examination:

HPV test A clinical study was run to study the efficacy of HPV tests. In patients whose Pap smears tested positive for mild abnormalities:

Between 50 and 80 patients in every 100 will test positive for HPV and will be advised to have a colposcopy and biopsy.

Between 20 and 50 patients in every 100 will test negative for HPV and need no further checks for 12 months.

Emily, the patient you read about who chose the HPV test, was a participant in this clinical trial.

Usual care (repeat Pap testing)

If a patient decides to follow usual care, the chance of having a colposcopy is lower. Between 20 and 40 out of every 100 patients who have a mildly abnormal Pap smear will be advised to have a colposcopy over a two year period. This is because many minor abnormalities clear up by themselves over time.

HPV - Negative Anecdote condition continued

What is a colposcopy?

A colposcopy is a more detailed examination of the cervix using an instrument called a colposcope, which is like a pair of binoculars on a stand.

The doctor will insert a speculum into the vagina to hold it open, as was done for the Pap smear.

The cervix is painted with a liquid which makes any abnormal areas change color.

The doctor looks through the colposcope to examine the cervix, to see the location and pattern of the abnormal areas.

During the colposcopy, a small sample of tissue, called a **biopsy**, may be taken from any abnormal looking areas of the cervix to confirm a diagnosis.

Timing

HPV testing gives patients the chance of having an earlier test result because they can arrange to have the test straight away. This means that:

If a more serious problem is detected any treatment can be carried out sooner.

Patients have a shorter period to wait for the test results. For some patients this means they experience less anxiety and worry.

In comparison, usual care can sometimes be a long process. Patients have to wait six months for their first follow up Pap smear and it takes over two years until they return to routine screening. This period of time can cause some patients to feel worried for a long time.

HPV - Negative Anecdote + Icon Array

Imagine you or a loved one is considering receiving the HPV test. You decide to go online to learn more information about the HPV test.

Now you'll read about Emily's experience receiving the HPV test.

"I was quite annoyed, couldn't really believe it that it happened to me. And because it was sexually transmitted, and because I haven't had a lot of partners. Yeah wasn't very happy at all really....I phoned the helpline and they reassured me, they really did reassure me so I felt quite happy after talking with them....I would say it [the anxiety] lessened over the year because of the chat I had with the helpline...I thought well if I hadn't taken part in that test I would never have known I had it and I knew I was going to have further tests."

HPV - Negative Anecdote + Icon Array continued

Now we will ask you to read some information about the HPV test.

A new test is available for patients with a mildly abnormal Pap smear. It is called the **HPV (Human papillomavirus) test**.

Patients can choose whether they would like the new test or the standard usual care for those with a mildly abnormal result, a repeat Pap smear in six months. Both managements are highly effective, however, each has its own advantages and disadvantages.

What is a Pap smear?

The Pap smear checks for changes in the cells at the neck of the womb (the cervix) at the top of the vagina.

It detects early changes in the cells which if left untreated may become cancer.

Most cancers of the cervix take up to 10 years to develop.

When a Pap smear is performed, a small sample of cells is taken from the cervix and is examined under a microscope.

A mildly abnormal Pap result means that very slight changes in the cells have been found. The cause of the cell changes is often not clear. They are commonly caused by the presence of **HPV**.

[See Figure 6 for corresponding image shown to participants.]

What is HPV?

HPV is one of the most common viral infections. It is thought that over 70% of women will have HPV at some stage during their lifetime.

Recent studies have shown that most cases of cervical cancer are caused by HPV.

The abnormal cells changes **ARE NOT** cancer.

It is important to remember that medical care for abnormal cells of the cervix is **very effective**.

Options for follow-up examination:

<u>HPV test</u> A clinical study was run to study the efficacy of HPV tests. In patients whose Pap smears tested positive for mild abnormalities:

Between 50 and 80 patients in every 100 will test positive for HPV and will be advised to have a colposcopy and biopsy.

Between 20 and 50 patients in every 100 will test negative for HPV and need no further checks for 12 months.

Emily, the patient you read about who chose the HPV test, was a participant in this clinical trial.

<u>Usual care (repeat Pap testing)</u>

If a patient decides to follow usual care, the chance of having a colposcopy is lower. Between 20 and 40 out of every 100 patients who have a mildly abnormal Pap smear will be advised to have a colposcopy over a two year period. This is because many minor abnormalities clear up by themselves over time.

HPV - Negative Anecdote + Icon Array continued

What is a colposcopy?

A colposcopy is a more detailed examination of the cervix using an instrument called a colposcope, which is like a pair of binoculars on a stand.

The doctor will insert a speculum into the vagina to hold it open, as was done for the Pap smear.

The cervix is painted with a liquid which makes any abnormal areas change color.

The doctor looks through the colposcope to examine the cervix, to see the location and pattern of the abnormal areas.

During the colposcopy, a small sample of tissue, called a **biopsy**, may be taken from any abnormal looking areas of the cervix to confirm a diagnosis.

Imagine 100 people like you sitting in a cinema. Now, imagine they all choose the usual care, a repeated Pap test. Out of those 100 people, 20-40 will be recommended to have a colposcopy examination over a two year period.

Now, imagine they all choose the HPV test. Out of those 100 people, 50-80 will be recommended to have a colposcopy.

[See Figure 7 for the icon array.]

Timing

HPV testing gives patients the chance of having an earlier test result because they can arrange to have the test straight away. This means that:

If a more serious problem is detected any treatment can be carried out sooner.

Patients have a shorter period to wait for the test results. For some patients this means they experience less anxiety and worry.

In comparison, usual care can sometimes be a long process. Patients have to wait six months for their first follow up Pap smear and it takes over two years until they return to routine screening. This period of time can cause some patients to feel worried for a long time.

IUD - Statistics condition

Imagine you or a loved one is considering getting a hormonal IUD. You decide to go online to learn more information about hormonal IUDs.

Now we will ask you to read some information about hormonal IUDs (intrauterine devices).

One birth control option patients can choose from is called the hormonal **IUD (intrauterine device)**.

Key points about hormonal IUDs:

Long-term pregnancy protection. Very effective for 5 years, immediately reversible.

Inserted into the uterus by a specifically trained provider.

Little required of the client once in place.

IUD - Statistics condition continued

Bleeding changes are common but not harmful. Typically, lighter and fewer days of bleeding, or infrequent or irregular bleeding.

What is a hormonal IUD?

T-shaped plastic piece that is placed in the uterus.

Steadily releases small amounts of the hormone Levonorgestrel (a progestin widely used in implants and oral contraceptive pills).

Works primarily by suppressing the growth of the lining of the uterus.

[See Figure 8 for the corresponding image presented to participants]

How effective is it?

One of the most *effective* and *long-lasting* methods.

In a clinical trial studying the efficacy of the hormonal IUD in preventing pregnancy, there was less than 1 pregnancy per 100 women using an IUD over the first year (2 per 1,000 women). This means that 998 of every 1,000 women using hormonal IUDs will not become pregnant.

A small risk of pregnancy remains beyond the first year of use and continues as long as the woman is using the IUD.

Over 5 years of hormonal IUD use: less than 1 pregnancy per 100 women (5 to 8 per 1,000 women).

Potential side effects:

Changes in bleeding patterns, including:

- Lighter bleeding and fewer days of bleeding
- Infrequent bleeding
- Irregular bleeding
- No monthly bleeding
- Prolonged bleeding

Acne

Headaches

Breast tenderness or pain

Nausea

Weight gain

Dizziness

Mood changes

Other possible changes include ovarian cysts.

[See Figure 9 for the corresponding image participants were shown.]

IUD - Statistics condition continued
**Complications**
Rare:
Puncturing (perforation) of the wall of the uterus by the IUD or an instrument used for insertion. Usually heals without treatment.
Very rare:
Miscarriage, preterm birth, or infection in the very rare case that the woman becomes pregnant with the IUD in place.
**What other birth control methods can I choose from?**
Sterilization
Implant
Birth control shot
Birth control ring
Birth control patch
Birth control pills
Condoms
Natural family planning

IUD - Statistics + Icon Array
Imagine you or a loved one is considering getting a hormonal IUD. You decide to go online to learn more information about hormonal IUDs.
Now we will ask you to read some information about hormonal IUDs (intrauterine devices).
One birth control option patients can choose from is called the hormonal **IUD (intrauterine device)**.
**Key points about hormonal IUDs:**
**Long-term pregnancy protection.** Very effective for 5 years, immediately reversible.
Inserted into the uterus by a specifically trained provider.
Little required of the client once in place.
**Bleeding changes are common but not harmful.** Typically, lighter and fewer days of bleeding, or infrequent or irregular bleeding.
**What is a hormonal IUD?**
T-shaped plastic piece that is placed in the uterus.
Steadily releases small amounts of the hormone Levonorgestrel (a progestin widely used in implants and oral contraceptive pills).
Works primarily by suppressing the growth of the lining of the uterus.
[See Figure 8 for the corresponding image presented to participants]

IUD - Statistics + Icon Array continued

How effective is it?

One of the most *effective* and *long-lasting* methods.

In a clinical trial studying the efficacy of the hormonal IUD in preventing pregnancy, there was less than 1 pregnancy per 100 women using an IUD over the first year (2 per 1,000 women). This means that 998 of every 1,000 women using hormonal IUDs will not become pregnant.

A small risk of pregnancy remains beyond the first year of use and continues as long as the woman is using the IUD.

Over 5 years of hormonal IUD use: less than 1 pregnancy per 100 women (5 to 8 per 1,000 women).

Imagine 100 people like you sitting in a cinema. Now, imagine they all choose a hormonal IUD for birth control. Out of those 100 people, the IUD will have effectively prevented pregnancy for 99 people.

[See Figure 10 for corresponding Icon Array.]

Potential side effects:

Changes in bleeding patterns, including:

- Lighter bleeding and fewer days of bleeding
- Infrequent bleeding
- Irregular bleeding
- No monthly bleeding
- Prolonged bleeding

Acne

Headaches

Breast tenderness or pain

Nausea

Weight gain

Dizziness

Mood changes

Other possible changes include ovarian cysts.

[See Figure 9 for the corresponding image participants were shown.]

Complications

Rare:

Puncturing (perforation) of the wall of the uterus by the IUD or an instrument used for insertion. Usually heals without treatment.

Very rare:

Miscarriage, preterm birth, or infection in the very rare case that the woman becomes pregnant with the IUD in place.

IUD - Statistics + Icon Array continued

What other birth control methods can I choose from?

Sterilization

Implant

Birth control shot

Birth control ring

Birth control patch

Birth control pills

Condoms

Natural family planning

IUD - Negative Anecdote condition

Imagine you or a loved one is considering getting a hormonal IUD. You decide to go online to learn more information about hormonal IUDs.

Now you'll read about Jennifer's experience having a hormonal IUD inserted.

"I got a Jaydess iud, and man what a relief to remove it. Insertion was horrible, and the pains didn't stop. I used painkillers for two months, and had spotting/light bleeding for those months. I tried for 7 months before giving up. It made me feel extremely depressed and anxious. When my lady doc pulled it out, it was like all my problems dissapeared with it. But it works out fine for some, and not for some. So don't knock it till you try it."

Now we will ask you to read some information about hormonal IUDs (intrauterine devices).

One birth control option patients can choose from is called the hormonal **IUD (intrauterine device)**.

Key points about hormonal IUDs:

Long-term pregnancy protection. Very effective for 5 years, immediately reversible.

Inserted into the uterus by a specifically trained provider.

Little required of the client once in place.

Bleeding changes are common but not harmful. Typically, lighter and fewer days of bleeding, or infrequent or irregular bleeding.

What is a hormonal IUD?

T-shaped plastic piece that is placed in the uterus.

Steadily releases small amounts of the hormone Levonorgestrel (a progestin widely used in implants and oral contraceptive pills).

Works primarily by suppressing the growth of the lining of the uterus.

[See Figure 8 for the corresponding image presented to participants]

IUD - Negative Anecdote condition continued

How effective is it?

One of the most *effective* and *long-lasting* methods.

In a clinical trial studying the efficacy of the hormonal IUD in preventing pregnancy, there was less than 1 pregnancy per 100 women using an IUD over the first year (2 per 1,000 women). This means that 998 of every 1,000 women using hormonal IUDs will not become pregnant.

Jennifer, the patient you read about who chose to have a hormonal IUD inserted, was a participant in this clinical trial.

A small risk of pregnancy remains beyond the first year of use and continues as long as the woman is using the IUD.

Over 5 years of hormonal IUD use: less than 1 pregnancy per 100 women (5 to 8 per 1,000 women).

Potential side effects:

Changes in bleeding patterns, including:

- Lighter bleeding and fewer days of bleeding
- Infrequent bleeding
- Irregular bleeding
- No monthly bleeding
- Prolonged bleeding

Acne

Headaches

Breast tenderness or pain

Nausea

Weight gain

Dizziness

Mood changes

Other possible changes include ovarian cysts.

[See Figure 9 for the corresponding image participants were shown.]

Complications

Rare:

Puncturing (perforation) of the wall of the uterus by the IUD or an instrument used for insertion. Usually heals without treatment.

Very rare:

Miscarriage, preterm birth, or infection in the very rare case that the woman becomes pregnant with the IUD in place.

IUD - Negative Anecdote continued
**What other birth control methods can I choose from?**
Sterilization
Implant
Birth control shot
Birth control ring
Birth control patch
Birth control pills
Condoms
Natural family planning

IUD - Negative Anecdote + Icon Array
Imagine you or a loved one is considering getting a hormonal IUD. You decide to go online to learn more information about hormonal IUDs.
Now you'll read about Jennifer's experience having a hormonal IUD inserted.
"I got a Jaydess iud, and man what a relief to remove it. Insertion was horrible, and the pains didn't stop. I used painkillers for two months, and had spotting/light bleeding for those months. I tried for 7 months before giving up. It made me feel extremely depressed and anxious. When my lady doc pulled it out, it was like all my problems dissapeared with it. But it works out fine for some, and not for some. So don't knock it till you try it."
Now we will ask you to read some information about hormonal IUDs (intrauterine devices).
One birth control option patients can choose from is called the hormonal **IUD (intrauterine device)**.
**Key points about hormonal IUDs:**
**Long-term pregnancy protection.** Very effective for 5 years, immediately reversible.
Inserted into the uterus by a specifically trained provider.
Little required of the client once in place.
**Bleeding changes are common but not harmful.** Typically, lighter and fewer days of bleeding, or infrequent or irregular bleeding.
**What is a hormonal IUD?**
T-shaped plastic piece that is placed in the uterus.
Steadily releases small amounts of the hormone Levonorgestrel (a progestin widely used in implants and oral contraceptive pills).
Works primarily by suppressing the growth of the lining of the uterus.
[See Figure 8 for the corresponding image presented to participants]

IUD - Negative Anecdote + Icon Array continued

How effective is it?

One of the most *effective* and *long-lasting* methods.

In a clinical trial studying the efficacy of the hormonal IUD in preventing pregnancy, there was less than 1 pregnancy per 100 women using an IUD over the first year (2 per 1,000 women). This means that 998 of every 1,000 women using hormonal IUDs will not become pregnant.

Jennifer, the patient you read about who chose to have a hormonal IUD inserted, was a participant in this clinical trial.

A small risk of pregnancy remains beyond the first year of use and continues as long as the woman is using the IUD.

Over 5 years of hormonal IUD use: less than 1 pregnancy per 100 women (5 to 8 per 1,000 women).

Imagine 100 people like you sitting in a cinema. Now, imagine they all choose a hormonal IUD for birth control. Out of those 100 people, the IUD will have effectively prevented pregnancy for 99 people.

[See Figure 10 for corresponding Icon Array.]

Potential side effects:

Changes in bleeding patterns, including:

  Lighter bleeding and fewer days of bleeding

  Infrequent bleeding

  Irregular bleeding

  No monthly bleeding

  Prolonged bleeding

Acne

Headaches

Breast tenderness or pain

Nausea

Weight gain

Dizziness

Mood changes

Other possible changes include ovarian cysts.

[See Figure 9 for the corresponding image participants were shown.]

Complications

Rare:

Puncturing (perforation) of the wall of the uterus by the IUD or an instrument used for insertion. Usually heals without treatment.

Very rare:

Miscarriage, preterm birth, or infection in the very rare case that the woman becomes pregnant with the IUD in place.

IUD - Negative Anecdote + Icon Array continued
**What other birth control methods can I choose from?**
Sterilization
Implant
Birth control shot
Birth control ring
Birth control patch
Birth control pills
Condoms
Natural family planning

Experiment 2 Supplemental Results

In addition to the models reported in the main text, we also evaluated a model which included icon arrays (present or absent) as a predictor in addition to anecdotes and sex. We fit a Bayesian multivariate cumulative model regressing efficacy attitudes on the presence of an Anecdote (Reference = No Anecdote), Sex (Reference = Female), Icon array (Reference = Absent), and the interaction between these predictors. There were no meaningful interactions with icon arrays and other predictors. Further, icon arrays did not materially affect participants' attitudes towards the efficacy of the treatment or their willingness to try it, $b_{\text{Effective}} = 0.10$, 95% CI [−0.22, 0.42]; $b_{\text{Try}} = 0.01$, 95% CI [−0.28, 0.31].

As an exploratory analysis, we wanted to examine whether age interacts with the anecdote and sex in how participants responded to each treatment. We fit a Bayesian multivariate cumulative model regressing efficacy attitudes on the presence of an Anecdote (Reference = No Anecdote), Sex (Reference = Female), Age (standardized), and the interaction between these predictors. There were no meaningful interactions or main effects involving age (see Table S12 for results).

Table S9 - Experiment 1c exploratory analysis involving Gender as a predictor

Parameter	*Estimate*	*Error*	*Lower*	*Upper*
Negative Anecdote (Effective)	–1.46	0.21	–1.86	–1.05
Icon Array (Effective)	1.07	0.21	0.66	1.48
Gender (Effective)	0.09	0.22	–0.33	0.51
Anecdote × Icon Array (Effective)	–0.21	0.28	–0.76	0.34
Anecdote × Gender (Effective)	–0.10	0.29	–0.66	0.47
Icon Array × Gender (Effective)	–0.18	0.30	–0.76	0.43
Anecdote × Icon Array × Gender (Effective)	0.54	0.40	–0.27	1.30
Negative Anecdote (Try)	–1.23	0.31	–1.85	–0.64
Icon Array (Try)	1.08	0.31	0.50	1.71
Gender (Try)	–0.07	0.32	–0.69	0.56
Anecdote × Icon Array (Try)	0.10	0.39	–0.70	0.86
Anecdote × Gender (Try)	0.02	0.42	–0.83	0.83
Icon Array × Gender (Try)	–0.52	0.42	–1.36	0.29
Anecdote × Icon Array × Gender (Try)	–0.29	0.53	–1.30	0.73
Negative Anecdote (Child)	–0.80	0.26	–1.31	–0.29
Icon Array (Child)	1.11	0.26	0.60	1.61
Gender (Child)	0.28	0.27	–0.25	0.83
Anecdote × Icon Array (Child)	–0.32	0.34	–0.99	0.36
Anecdote × Gender (Child)	–0.20	0.36	–0.94	0.50
Icon Array × Gender (Child)	–0.67	0.36	–1.36	0.03
Anecdote × Icon Array × Gender (Child)	0.07	0.47	–0.84	0.97

Note: The No Anecdote condition, no Icon Array, and Gender (Female) were the reference groups in this analysis. Lower and Upper indicate the 95% credible intervals for each estimate. Threshold and group-level parameters are omitted for brevity.

Table S10 - Experiment 1c exploratory analysis involving Age as a predictor

Parameter	*Estimate*	*Error*	*Lower*	*Upper*
Negative Anecdote (Effective)	−1.48	0.17	−1.82	−1.15
Icon Array (Effective)	1.01	0.17	0.68	1.35
Age (Effective)	−0.17	0.12	−0.41	0.07
Anecdote × Icon Array (Effective)	0.01	0.23	−0.45	0.45
Anecdote × Age (Effective)	0.28	0.17	−0.05	0.62
Icon Array × Age (Effective)	0.27	0.16	−0.05	0.59
Anecdote × Icon Array × Age (Effective)	−0.22	0.23	−0.68	0.24
Negative Anecdote (Try)	−1.20	0.26	−1.72	−0.70
Icon Array (Try)	0.90	0.27	0.38	1.43
Age (Try)	−0.34	0.19	−0.72	0.04
Anecdote × Icon Array (Try)	−0.12	0.35	−0.82	0.57
Anecdote × Age (Try)	0.51	0.27	−0.01	1.03
Icon Array × Age (Try)	0.23	0.26	−0.27	0.73
Anecdote × Icon Array × Age (Try)	0.22	0.35	−0.46	0.92
Negative Anecdote (Child)	−0.86	0.22	−1.30	−0.43
Icon Array (Child)	0.88	0.22	0.46	1.32
Age (Child)	−0.19	0.16	−0.51	0.13
Anecdote × Icon Array (Child)	−0.35	0.30	−0.94	0.22
Anecdote × Age (Child)	0.15	0.23	−0.30	0.59
Icon Array × Age (Child)	−0.06	0.21	−0.49	0.35
Anecdote × Icon Array × Age (Child)	0.27	0.30	−0.31	0.86

Note: The No Anecdote condition and no Icon Array were the reference groups in this analysis. Age was standardized. Lower and Upper indicate the 95% credible intervals for each estimate. Threshold and group-level parameters are omitted for brevity.

Table S11 - Experiment 1c exploratory analysis involving Holistic Beliefs as a predictor

Parameter	*Estimate*	*Error*	*Lower*	*Upper*
Negative Anecdote (Effective)	−1.38	0.42	−2.22	−0.55
Icon Array (Effective)	1.02	0.43	0.18	1.87
Holistic Beliefs (Effective)	0.10	0.09	−0.08	0.28
Anecdote × Icon Array (Effective)	0.47	0.57	−0.62	1.56
Anecdote × Holistic Beliefs (Effective)	−0.03	0.11	−0.25	0.19
Icon Array × Holistic Beliefs (Effective)	0.00	0.11	−0.21	0.23
Anecdote × Icon Array × Holistic Beliefs (Effective)	−0.13	0.15	−0.43	0.17
Negative Anecdote (Try)	−0.66	0.56	−1.77	0.43
Icon Array (Try)	0.75	0.56	−0.35	1.85
Holistic Beliefs (Try)	0.02	0.14	−0.25	0.28
Anecdote × Icon Array (Try)	−0.51	0.66	−1.84	0.75
Anecdote × Holistic Beliefs (Try)	−0.18	0.16	−0.49	0.13
Icon Array × Holistic Beliefs (Try)	0.03	0.15	−0.27	0.33
Anecdote × Icon Array × Holistic Beliefs (Try)	0.14	0.19	−0.22	0.51
Negative Anecdote (Child)	−0.42	0.50	−1.41	0.54
Icon Array (Child)	0.71	0.49	−0.26	1.67
Holistic Beliefs (Child)	0.06	0.11	−0.15	0.28
Anecdote × Icon Array (Child)	0.01	0.60	−1.18	1.19
Anecdote × Holistic Beliefs (Child)	−0.13	0.14	−0.40	0.13
Icon Array × Holistic Beliefs (Child)	0.04	0.13	−0.22	0.30
Anecdote × Icon Array × Holistic Beliefs (Child)	−0.08	0.17	−0.41	0.25

Note: The No Anecdote condition and no Icon Array were the reference groups in this analysis. The Holistic Beliefs measure was the median of responses from the scale items. Lower and Upper indicate the 95% credible intervals for each estimate. Threshold and group-level parameters are omitted for brevity.

Table S12 - Experiment 1c exploratory analysis involving Medical Skepticism as a predictor

Parameter	*Estimate*	*Error*	*Lower*	*Upper*
Negative Anecdote (Effective)	−1.16	0.53	−2.21	−0.11
Icon Array (Effective)	0.07	0.54	-1.01	1.16
Medical Skepticism (Effective)	−0.07	0.11	−0.28	0.15
Anecdote × Icon Array (Effective)	0.50	0.64	−0.74	1.79
Anecdote × Medical Skepticism (Effective)	−0.08	0.13	−0.34	0.17
Icon Array × Medical Skepticism (Effective)	0.24	0.13	−0.02	0.49
Anecdote × Icon Array × Medical Skepticism (Effective)	−0.12	0.16	−0.43	0.18
Negative Anecdote (Try)	−0.89	0.68	−2.22	0.43
Icon Array (Try)	0.41	0.69	−0.93	1.77
Medical Skepticism (Try)	−0.18	0.16	−0.50	0.15
Anecdote × Icon Array (Try)	−0.02	0.73	−1.48	1.39
Anecdote × Medical Skepticism (Try)	−0.09	0.17	−0.42	0.24
Icon Array × Medical Skepticism (Try)	0.13	0.17	−0.22	0.46
Anecdote × Icon Array × Medical Skepticism (Try)	−0.02	0.19	−0.39	0.36
Negative Anecdote (Child)	−0.53	0.59	−1.67	0.65
Icon Array (Child)	0.03	0.64	−1.20	1.29
Medical Skepticism (Child)	−0.45	0.14	−0.72	−0.18
Anecdote × Icon Array (Child)	−1.05	0.69	−2.43	0.29
Anecdote × Medical Skepticism (Child)	−0.11	0.14	−0.39	0.17
Icon Array × Medical Skepticism (Child)	0.20	0.15	−0.10	0.50
Anecdote × Icon Array × Medical Skepticism (Child)	0.19	0.18	−0.16	0.55

Note: The No Anecdote condition and no Icon Array were the reference groups in this analysis. The Medical Skepticism measure was the median of responses from the scale items. Lower and Upper indicate the 95% credible intervals for each estimate. Threshold and group-level parameters are omitted for brevity.

Table 12

Table S12 - Experiment 2 exploratory analysis involving Age as a predictor

Parameter	*Estimate*	*Error*	*Lower*	*Upper*
**Negative Anecdote (Effective)**	**–0.31**	**0.12**	**–0.54**	**–0.07**
Sex (Effective)	–0.09	0.13	–0.34	0.15
Age (Effective)	0.15	0.08	–0.02	0.32
Anecdote × Sex (Effective)	0.25	0.18	–0.08	0.59
Anecdote × Age (Effective)	–0.11	0.12	–0.33	0.12
Sex × Age (Effective)	–0.12	0.12	–0.37	0.12
Anecdote × Sex × Age (Effective)	0.08	0.17	–0.24	0.41
**Negative Anecdote (Try)**	**–0.24**	**0.10**	**–0.43**	**–0.04**
Sex (Try)	0.02	0.10	–0.18	0.22
Age (Try)	0.01	0.07	–0.13	0.15
Anecdote × Sex (Try)	0.07	0.15	–0.21	0.36
Anecdote × Age (Try)	0.00	0.10	–0.20	0.19
Sex × Age (Try)	0.01	0.10	–0.19	0.30
Anecdote × Sex × Age (Try)	0.02	0.14	–0.26	0.30

Note: The No Anecdote condition and sex (Female) were the reference groups in this analysis. Lower and Upper indicate the 95% credible intervals for each estimate. Threshold and group-level parameters are omitted for brevity.

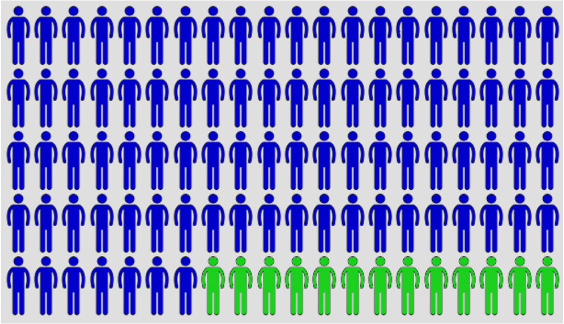

Figure 1. This is the icon array used in the icon array only condition in Experiment 1c. This image is a depiction of the effectiveness of B12 as a medical treatment. Imagine 100 people received B12 shots. The blue figures represent participants that would benefit from the B12 shots. The green figures represent participants who would fail to benefit from the B12 shots.

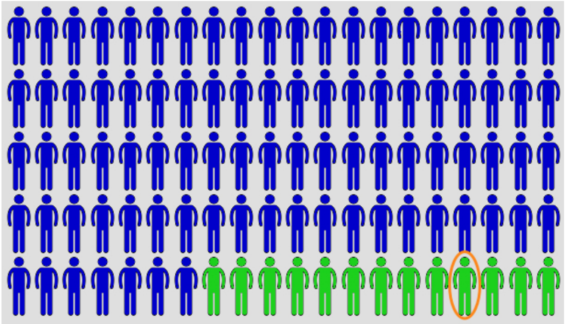

Figure 2. This is the icon array used in the icon array + negative anecdote condition in Experiment 1c. This image is a depiction of the effectiveness of B12 as a medical treatment. Imagine 100 people received B12 shots. The blue figures represent participants that would benefit from the B12 shots. The green figures represent participants who would fail to benefit from the B12 shots.

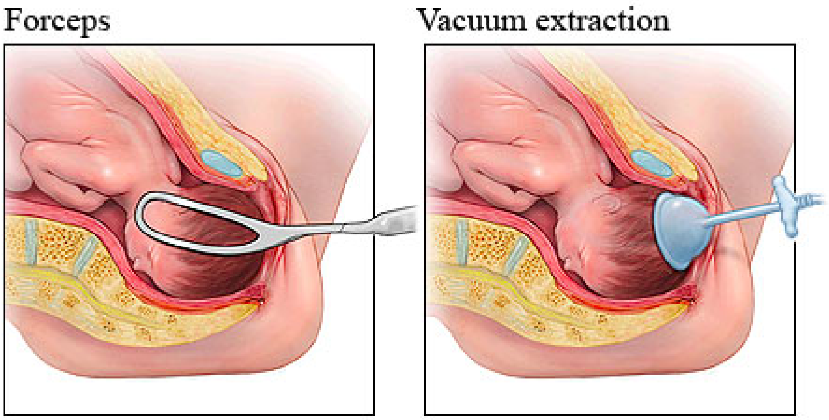

Figure 3. This image is a depiction of two different assisted delivery methods using either forceps or vacuum extraction.

	Have an epidural	Do not have an epidural
**What is usually involved?**	• A large needle is used to place a small tube, called a catheter, in your lower back. The needle is removed, and the catheter is taped to your skin.<br>• The medicine leaves you partly or completely numb below the waist, depending on how much is used.<br>• You will probably have to stay in bed and have your bladder emptied with a urinary catheter.<br>• You can probably move your legs and feel when it's time to push. But pushing may be harder.	• You use other ways to control pain, such as pain medicines or natural methods like breathing techniques, massage, and distraction.
**What are the benefits?**	• An epidural works very well to relieve pain.<br>• The medicine doesn't go into your bloodstream, so you remain awake and alert throughout labor and delivery.	• You avoid the risks and side effects of an epidural.<br>• Without pain medicine, you have a natural childbirth.
**What are the risks and side effects?**	• The risks of an epidural include:<br>• A drop in blood pressure.<br>• Being too numb to push and needing an assisted delivery.<br>• Having the baby move into the wrong position.<br>• The possible side effects include:<br>• A sore back.<br>• A severe headache.	• Labor may be more painful.<br>• Too much pain can tire you out so much that you may need other methods to help you deliver.

Figure 4. This table shows the involvement, benefits, and risk of having an epidural vs. not having one.

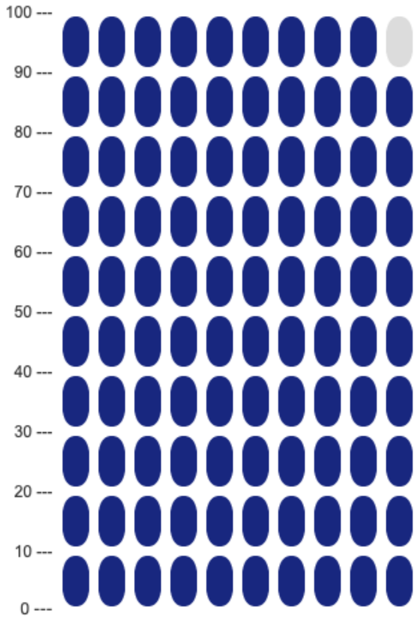

Figure 5. This is the icon array presented (along with the following text) to participants in the Stats + Icon Array and Icon Array + Negative Anecdote conditions.

"Imagine 100 people like you sitting in a cinema. Now, imagine they all choose to receive an epidural to relieve birthing pain. Out of those 100 people, 1 woman will experience a severe headache as the results of spinal cord puncturing."

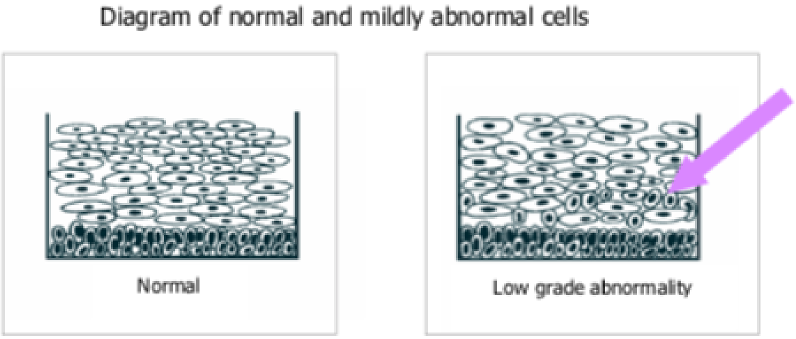

Figure 6. This image is a depiction of what mildly abnormal cells from a pap smear look like compared to normal ones.

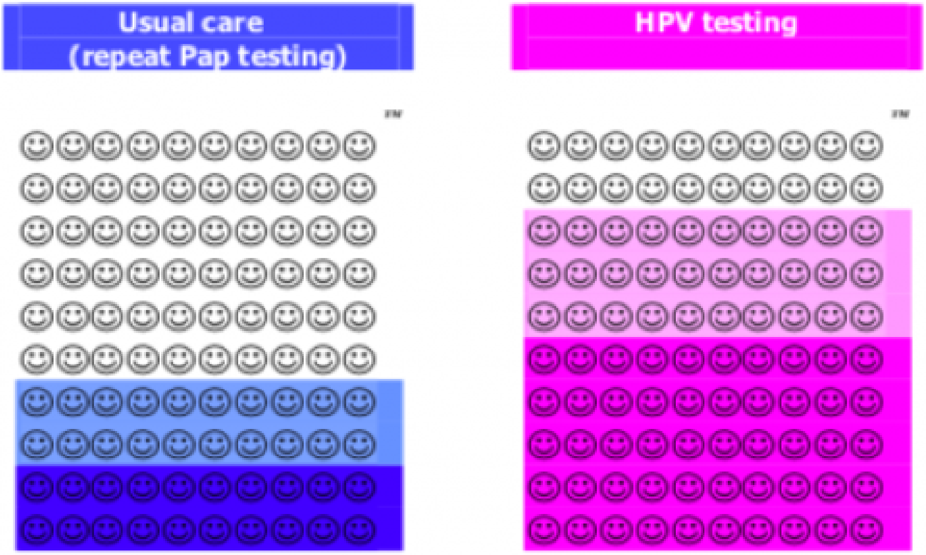

Figure 7. This is the icon array presented to participants along with the following text. “Imagine 100 people like you sitting in a cinema. Now, imagine they all choose the usual care, a repeated Pap test. Out of those 100 people, 20-40 will be recommended to have a colposcopy examination over a two year period. Now, imagine they all choose the HPV test. Out of those 100 people, 50-80 will be recommended to have a colposcopy.”

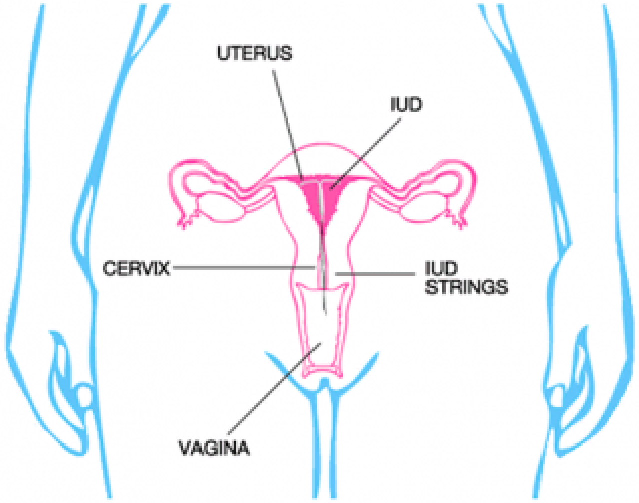

Figure 8. This image shows a diagram of where the IUD is placed.

**Known Health Benefits**	**Known Health Risks**
Helps protect against:<br>• Risks of pregnancy<br>• Iron-deficiency anemia<br>May help protect against:<br>• Pelvic inflammatory disease<br>Reduces:<br>• Menstrual cramps<br>• Symptoms of endometriosis	There are no known health risks

Figure 9. This image shows the associated health benefits and risks of getting an IUD.

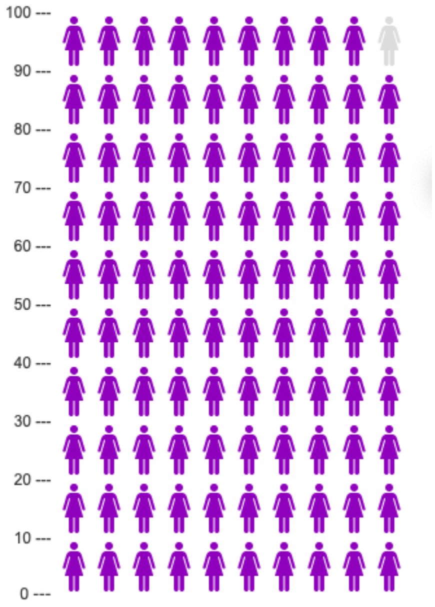

Figure 10. This is the icon array presented to participants along with the following text. “Imagine 100 people like you sitting in a cinema. Now, imagine they all choose a hormonal IUD for birth control. Out of those 100 people, the IUD will have effectively prevented pregnancy for 99 people.”
